# Genetic relatedness of previously Plant-Variety-Protected commercial maize inbreds

**DOI:** 10.1371/journal.pone.0189277

**Published:** 2017-12-13

**Authors:** Travis J. Beckett, A. Jason Morales, Klaus L. Koehler, Torbert R. Rocheford

**Affiliations:** 1 Department of Agronomy, Purdue University, West Lafayette, IN, United States of America; 2 Dow AgroSciences, Indianapolis, IN, United States of America; Institute of Genetics and Developmental Biology Chinese Academy of Sciences, CHINA

## Abstract

The emergence of high-throughput, high-density genotyping methods combined with increasingly powerful computing systems has created opportunities to further discover and exploit the genes controlling agronomic performance in elite maize breeding populations. Understanding the genetic basis of population structure in an elite set of materials is an essential step in this genetic discovery process. This paper presents a genotype-based population analysis of all maize inbreds whose Plant Variety Protection certificates had expired as of the end of 2013 (283 inbreds) as well as 66 public founder inbreds. The results provide accurate population structure information and allow for important inferences in context of the historical development of North American elite commercial maize germplasm. Genotypic data was obtained via genotyping-by-sequencing on 349 inbreds. After filtering for missing data, 77,314 high-quality markers remained. The remaining missing data (average per individual was 6.22 percent) was fully imputed at an accuracy of 83 percent. Calculation of linkage disequilibrium revealed that the average *r*^2^ of 0.20 occurs at approximately 1.1 Kb. Results of population genetics analyses agree with previously published studies that divide North American maize germplasm into three heterotic groups: Stiff Stalk, Non-Stiff Stalk, and Iodent. Principal component analysis shows that population differentiation is indeed very complex and present at many levels, yet confirms that division into three main sub-groups is optimal for population description. Clustering based on Nei’s genetic distance provides an additional empirical representation of the three main heterotic groups. Overall fixation index (*F*_ST_), indicating the degree of genetic divergence between the three main heterotic groups, was 0.1361. Understanding the genetic relationships and population differentiation of elite germplasm may help breeders to maintain and potentially increase the rate of genetic gain, resulting in higher overall agronomic performance.

## Introduction

**Fig 1:**
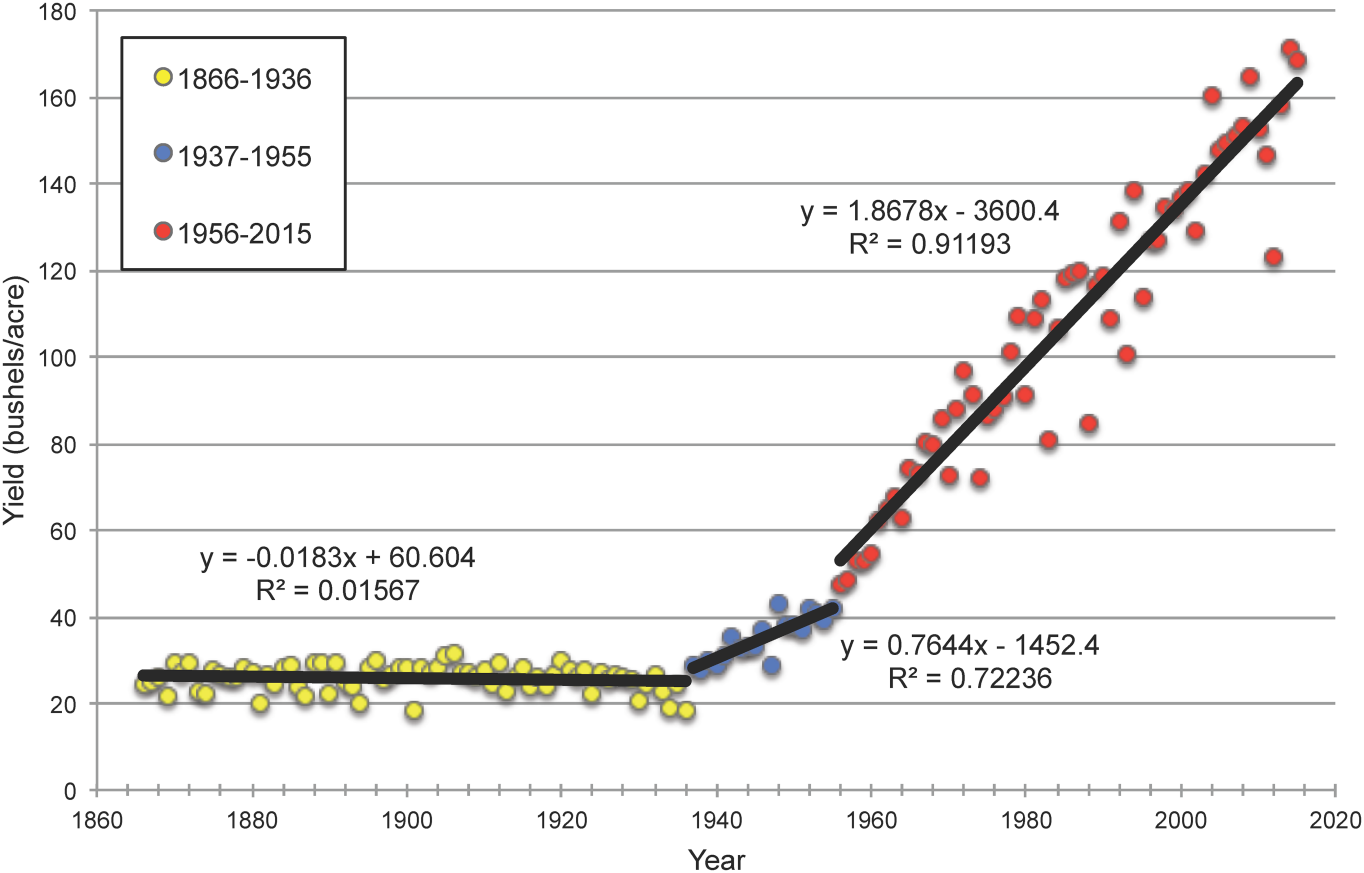
Historical U.S. Maize Yields, 1866 to 2015. Data is separated into three time periods according to the source of corn seed planted for agricultural production. In the first period, from 1866 to 1936, the vast majority of corn grown was of the open-pollinated type. During the second period, from 1937 to 1955, most hybrid corn planted in the U.S. was produced from double crosses. Throughout the third period, from 1956 to 2015, single-cross hybrids were the largest source of corn seed planted for commercial production. A best-fit linear trend is included for each time period. Data was obtained from the USDA National Agricultural Statistical Service [10].

Maize (*Zea mays* subsp. *mays*) is one of the most important agricultural crops in the United States. Grain yields remained generally constant from 1866 to 1936, when the vast majority of maize was grown from open-pollinated seeds (see Fig 1). Foundational studies in the early 1900’s on inbreeding and heterosis introduced the idea of producing commercial maize seed on a hybrid plant resulting from a cross of two inbreds [1–8]. Subsequently, the replacement of open-pollinated varieties with double- and single-cross hybrids played a major role in sustained increases in grain yield since 1937 [8, 9].

After over three decades of widespread commercial hybrid maize production, the Plant Variety Protection Act (PVPA) was passed by the U.S. Congress in 1970 [11]. This law guaranteed intellectual property rights to developers of new plant varieties by prohibiting others from reproducing, selling, or importing any protected variety, for a period of 18 (presently 20) years [12]. New plant varieties may also be protected by U.S. patents. The legality of granting patents for plants was affirmed by rulings by the U.S. Supreme Court in *Diamond v. Chakrabarty* (1980) [13] and *J.E.M. Ag Supply v. Pioneer* (2001) [14], and by the U.S. Board of Patent Appeals and Inferences in Ex parte Hibbert (1985) [15–17]. Both utility patents and PVP certificates are effective forms of germplasm protection commonly used by U.S. private-sector soybean and maize breeders [18, 19].

When a PVP certificate issued for a maize inbred expires, and there is no active patent protecting the property, the inbred then enters the public domain and is provided free of charge by the United States Department of Agriculture (USDA). Many of these now-publicly available inbred lines have made a significant contribution to current commercial germplasm. By pedigree analysis, Mikel (2011) found that the top four progenitors (by genetic contribution) of 305 maize inbreds registered by PVP and/or utility patent between the years 2004 and 2008 were 3IIH6 (12.2%), B73 (11.7%), PH207 (9.5%), and PHG29 (9.4%) [20]. Three of these inbreds, 3IIH6, PH207, and PHG29, have expired PVP certificates. All four inbreds mentioned above are included found the population used in this study. Each inbred with a newly expired PVP certificate is a readily available source of highly selected alleles and haplotype blocks that can likely improve germplasm pools in breeding programs that previously did not have access to such elite genetics [20].

Heterosis, also known as hybrid vigor, is observed when the F1 progeny of a cross between two individuals from different germplasm groups performs better than the F1 progeny of a cross between two individuals from the same germplasm group [21]. In maize, these germplasm groups are generally referred to as heterotic groups. Numerous proposals of North American maize heterotic group divisions have been made [21–26]. Most are some variant of the dominant heterotic pattern of Stiff Stalk (SS) and Non-Stiff Stalk (NSS), commonly known as the female-male heterotic pattern. A representative summary of the heterotic group proposals is given in Table 1.

**Table 1:** Summary of proposed heterotic group divisions in maize.

Author(s)	Year	Proposed heterotic group division
Troyer [22]	1999	Reid Yellow Dent, Minnesota 13, Northwestern Dent, Lancaster Sure Crop, and Leaming Corn
Gethi, et al. [23]	2002	Reid Yellow Dent, Lancaster, and other sub-groups
Mikel, et al. [24]	2006	Seven key ancestral inbreds: B73, Mo17, PH207, PHG39 (from B37), LH123Ht, LH82, and PH595
Nelson, et al. [25]	2008	B73, Mo17, PH207, A632, Oh43, B37, and mixed
Lu, et. al. [26]	2009	Iowa Stiff-Stalk Synthetic (BSSS) and non-Iowa Stiff-Stalk Synthetic (non-BSSS)
Bernardo [21]	2014	BSSS (B14, B37, B73) and non-BSSS (Iodent, Oh43, Mo17, and other subgroups)

Maize inbreds can be assigned to heterotic groups based on: (1) pedigree information and specific combining ability based on field trials; (2) molecular markers and genetic relatedness analysis; or (3) some combination of these two methods [27]. Many attempts to classify public maize lines into heterotic groups using molecular markers have been reported, with varying levels of success (See Table 2). Early molecular marker platforms produced small number of markers at inconsistent accuracy levels [28–34]. One problem with using a small number of markers is that it can be difficult to precisely resolve the heterotic and family group membership of closely related inbred lines, as the marker set may not include all loci that are responsible for heterotic divergence. Consequently, genetic-based determination of heterotic groups and combining ability was not considered as effective as traditional field-validation at accurately identifying similar groups of germplasm out of a large group of seemingly unrelated inbred lines [29, 34]. Genotyping technology has now improved to the point where genotype-based heterotic groupings appear just as accurate as the groupings defined by pedigrees and empirical field measures of combining ability [37, 40]. Next-generation sequencing methods such as genotyping-by-sequencing (GBS) can be very helpful in determining the heterotic group position of newly released ex-PVP inbreds relative to a breeding program’s existing inbreds.

**Table 2:** Using molecular markers to identify heterotic groups in maize.

Author(s) and year	No. of Inbreds	Population description	—Markers—	
			No.	Type
Smith, et al. (1990) [28]	37	North American temperate	257	RFLP
Dudley, et al. (1991) [29]	14	North American temperate	52	RFLP
Livini, et al. (1991) [30]	40	Italian temperate	149	RFLP
Melchinger, et al. (1991) [31]	32	North American temperate	83	RFLP
Mumm & Dudley (1994) [32]	148	North American temperate	46	RFLP
Senior, et al. (1998) [33]	94	North American temperate	70	SSR
Barata & Carena (2006) [34]	40	North American temperate	49	SSR
Nelson, et al. (2008) [25]	109	North American temperate^a^	614	SNP
Lu, et al. (2009) [26]	770	Tropical and temperate from CIMMYT, China, and Brazil	449	SNP
Kahler, et al. (2010) [37]	98	North American temperate^a^	285	SSR
van Heerwaarden, et al. (2012) [38]	294	North American temperate^a^	45,997	SNP
Olmos et al. (2013) [35]	103	Argentinean temperate	50	SSR
Romay et al. (2013) [39]	2,185	Global tropical and temperate^a^	681,257	SNP
Unterseer, et al. (2014) [40]	315	Global tropical and temperate	609,442	SNP
Smith, et al. (2015) [36]	380	North American temperate and sub-tropical^a^	635	SNP
Wu, et al. (2016) [41]	544	CIMMYT inbreds	362,008	SNP
Zhang, et al. (2016) [42]	362	Chinese tropical and temperate	56,110	SNP

^a^Includes inbreds with expired Plant Variety Protection certificates.

There are some challenges, however, presented by the GBS method. The success of GBS depends on a minimum read depth, or number of repeated sequences covering a specific locus. Read depth can vary across the genome, between separate GBS batches, and even between individuals [43]. Due to low coverage of sequencing, there may be large portions of the genome without any successful marker calls [44]. Therefore, each set of GBS data–and even each individual genotype–has a unique distribution of the number and quality of genotype calls. Fortunately, when missing data remains after filtering, it can usually be imputed at acceptable levels of accuracy–a very cost-favorable alternative to sequencing at a higher depth [43, 45, 46].

Following the development of next-generation sequencing platforms, there have been a number of studies published on genetic classification of maize inbred lines [25, 26, 36–42]. Several included inbreds with expired Plant Variety Protection certificates: Nelson, et al.(2008) with 92 ex-PVP inbreds; Kahler, et al. (2010) with 33; van Heerwaarden, et al. (2012) with 137; Romay, et al. (2015), with 212; and Smith, et al. (2015) with 105. Out of these, the publication most closely aligned to the subject of this study is that authored by Romay et al. (2013) [38].

This paper presents a comprehensive genotype-based population analysis of all ex-PVP maize inbreds available as of the end of 2012. The robust array of analyses includes measures of genetic diversity, linkage disequilibrium, genotypic clustering, and heterotic groupings. Included in this study is a greater number of ex-PVP inbreds (283) and a wider range of analytical methods than found in previous publications. The results herein can help maize breeders determine how to best incorporate the ex-PVP inbreds into their existing germplasm pools.

## Materials and methods

### Plant material

**Fig 2:**
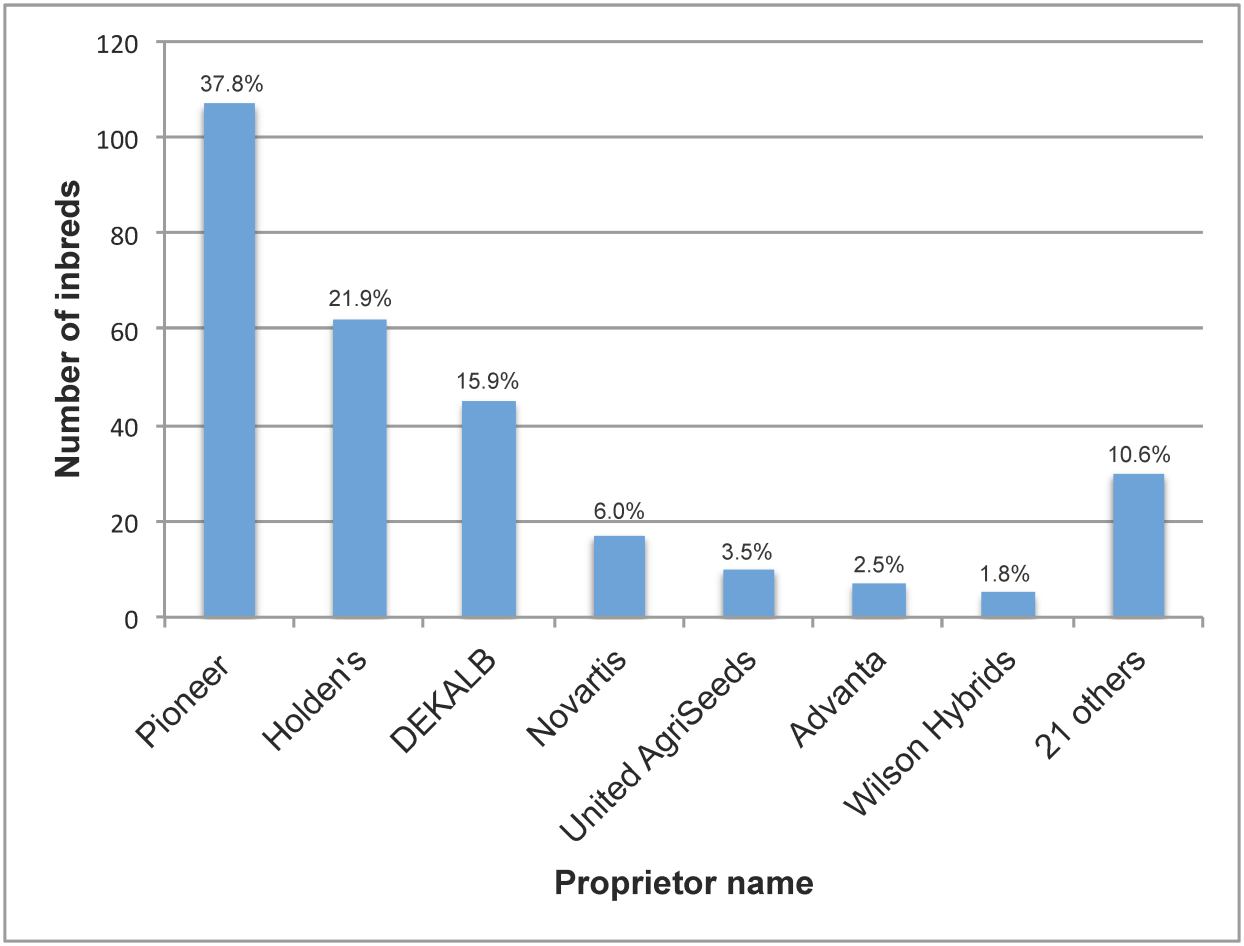
Plant Variety Protection certificates expired as of 2012, by proprietor. Proprietor names were abbreviated as follows: Pioneer, Pioneer Hi-Bred International, Inc.; Holden’s, Holden’s Foundation Seeds, Inc.; DEKALB, DEKALB Genetics; Novartis, Novartis Seeds, Inc; United AgriSeeds, United AgriSeeds, Inc.; Advanta, Advanta Technology Limited; and Wilson Hybrids, Wilson Hybrids Inc. Proprietor names are on the x-axis, and the number of inbreds present in this set of 283 is on the y-axis. Above each bar is the value in percent, calculated as the number of PVP inbreds for each respective proprietor divided by 283. Proprietorship was obtained from the Plant Variety Protection certificate for each inbred, accessible in the United States Department of Agriculture Agricultural Research Service Germplasm Network Information Database [48].

The maize varieties used in this study include 283 ex-PVP inbreds and 66 public inbred founders. The 283 ex-PVP inbreds were those with certificates that had expired between 1994 and 2012. Distribution by proprietor of these 283 inbreds with expired Plant Variety Protection (ex-PVP) is shown in Fig 2. Pedigrees of the 283 ex-PVP lines were examined and 66 public founder inbreds were identified based on two criteria: (1) the public inbred appeared in the pedigree of at least one ex-PVP inbred; and (2) seed for that public line was available at the start of this project [47]. Seed for all 349 inbreds was requested from the USDA-ARS National Genetic Resources Program [48], and received from the USDA-ARS North-Central Regional Plant Introduction Station (NCRPIS) in Ames, Iowa. Ex-PVP inbred pedigrees were obtained from the PVP certificates, accessed at ars.grin.gov [48]. Public inbred pedigrees were obtained from the volume titled, Compilation of North American Maize Breeding Germplasm [49]. Tables with general information about both the ex-PVP and public founder inbred sets are provided in the supplementary information (see S1 and S2 Tables).

A bar chart showing the distribution of the 283 ex-PVP inbreds used in this study, sorted by proprietor, is displayed in Fig 2. Pioneer Hi-Bred International, Inc. (Pioneer) produced the most inbreds, with nearly 40 percent of these PVP certificates. The top three proprietors, Pioneer, Holden’s Foundation Seeds, and DEKALB Plant Genetics, together held over 75 percent of PVP certificates for inbred lines used in this data set. The top seven proprietors, which also includes Novartis Seeds, Inc., United AgriSeeds, Inc., Advanta Technology Limited, and Wilson Hybrids, Inc., accounted for nearly 90 percent of PVP certificates. The remaining ex-PVP inbreds used in this study originated from twenty-one different companies, with between one and three certificates held by each company. Thus, of the North American commercial maize inbreds with PVP certificates that had expired as of the end of 2012, the vast majority (nearly 90%) came from only one-quarter of all private maize breeding programs that used PVP for their inbreds (seven out of twenty-eight companies).

### Genotypic data compilation

The original genotypic data comes from two sources. The first source includes genotyping data on 224 lines whose PVP certificates had expired as of the end of 2011, as well as 67 public founder inbred lines. Partially imputed GBS data for these 291 lines was downloaded from the online GBS data repository at www.panzea.org [50]. The build version was ZeaGBSv27, with 955,690 SNPs on AGPv2 coordinates, produced using the enzyme ApeKI and the protocol described by Elshire et al., (2011) [43, 51]. The second source consisted of GBS data on 58 additional ex-PVP inbred lines whose PVP certificates expired during the first four months of 2012. These 58 lines were grown at the Purdue Agronomy Center for Research and Education (ACRE) in West Lafayette, Indiana, in the summer of 2012. Tissue sampling and DNA extraction was performed according to the protocol of Romay et al., (2013) [38]. The DNA samples were sent to the Cornell University Institute for Genomic Diversity (Ithaca, New York), where GBS libraries were prepared and analyzed according to Elshire et al. (2011) [43], using the enzyme ApeKI for digestion and creating a library with 240,021,078 unique barcodes. The GBS pipeline for these 58 lines resulted in 546,531 unfiltered SNPs. The two genotypic data sets were aligned and merged, using TASSEL 5.0, version 20151210 [52]. A summary of the genotypic data set compilation steps is given in Table 3.

**Table 3:** Summary statistics of unmerged genotypic data sets, before filtering and imputing.

Statistic	GBS set no. 1^a^	GBS set no. 2^b^	Merged GBS set
Inbreds	291	58	349
Sites	955,690	546,531	1,281,671
Total data points^c^	278,105,790	31,698,798	447,303,179
Missing SNPs	36,217,717	13,724,629	187,442,397
Percent missing	13.0%	43.3%	41.9%
No. Heterozygous	502,897	596,243	1,100,366
Percentage het.	0.18%	1.88%	0.25%
Sites common to both GBS sets			220,550
Percent Sites common to both GBS sets^d^			17.2%

^a^ The data for these 291 inbreds was obtained from from the online GBS data repository at www.panzea.org [50].

^b^ The GBS data set for these 58 inbreds was produced by Cornell University Institute for Genomic Diversity (Ithaca, New York).

^c^ Total data points = Inbreds x Sites = Total number of SNP reads.

^d^ Calculated by (Sites common to both GBS sets) / (Merged Sites)

### Data analysis

#### SNP characteristics

Quality control measures were employed to ensure that the genotypic data would be as accurate as possible for population structure analysis. Genotypic markers with missing data greater than ten percent and/or a minor allele frequency (MAF) less than 0.05 were removed. As the genotypic analyses assume only two alleles per locus, minor SNP statuses (i.e. tertiary and greater alleles) were changed to missing data. Additionally, any heterozygote calls were changed to missing data. Applying these filters reduced the maximum amount of missing data per inbred to no more than 30 percent for any one inbred in this data set (see Table 4). The specific level of 30 percent was chosen to balance the share of missing data between the two previously unmerged GBS sets while also minimizing the proportion of overall missing data, thus reducing overall proportion of genotypic errors caused by imputation [53]. These filter thresholds left the genotypic data set with a total of 77,314 SNPs.

**Table 4:** Summary statistics of merged, filtered, and imputed genotypic data sets.

Statistic	Merged data^a^	Filtered data^b^	Imputed data^c^
Inbreds	349	349	349
Sites	1,281,671	77,314	77,314
Total data points^c^	447,303,179	26,987,123	26,987,123
Missing SNPs	187,442,397	1,680,218	0
Percent missing	41.9%	6.22%^d^	0
Heterozygous	1,100,366	0	0
Percentage het.	0.25%	0	0

^a^ Two genotyping-by-sequencing (GBS) data sets were merged for this study. One consisted of 546,531 SNP reads on 58 inbreds, and the other had 1,290,050 SNP reads on 291 inbreds.

^b^ Filtering consisted of: (1) removing markers with minor allele frequency (MAF) less than 0.05; (2) removing markers with greater than 17.2 percent missing data; and (3) changing the genotype call to missing at all heterozygous sites and all minor SNP states (tertiary and above).

^c^ Total data points = Inbreds x Sites = Total number of SNP reads.

^d^ For missing SNPs per inbred: median was 3.90%; maximum was 28.5%; and minimum was 0.66%.

The 6.22 percent of genotypic data points that remained as missing data were fully imputed using the ‘markov’ function in the package ‘NAM’ in RStudio version 0.98.1103 [53–55]. This function employs a Hidden Markov Model (HMM); however, unlike other HMM-based imputation methods, the ‘markov’ function only runs in the forward direction and not the backward direction. This feature enables quicker imputation computations for very large data sets. Imputation accuracy was calculated by comparing a completed genotypic data set with a version of the same data set which included imputed values at randomly placed missing data points. Calculations to assess imputation accuracy were repeated 100 times using the same complete data set, with the average amount of randomly placed missing data across the repetitions set at 6.22 percent. The mean imputation accuracy of these repetitions was reported as the overall imputation accuracy for this data set.

#### Principal component analysis

Principal component analysis (PCA, or PC analysis) was performed by the ‘prcomp’ function in RStudio [55]. The optimal number of PCAs was determined by consulting both the scree plot and the PCA plots, in context of what has already been reported about the number of major maize heterotic group divisions [21–26].

#### Linkage disequilibrium

Analysis of linkage disequilibrium (LD) was performed in RStudio [55] with the package ‘NAM’, using the function ‘ld’ [54]. Decay of LD was determined for each chromosome individually by considering all pairwise SNP marker combinations. For each SNP pair, both the distance (bp) and the coefficient of determination *r*^2^ were calculated, then plotted. A smoothing function within RStudio (’lokern’) was employed to insert a trend line for each chromosome [56]. A trend line for the mean LD over all chromosomes was also included in the plot.

#### Population structure

Population substructure was analyzed using RStudio [55], using various packages as described below. Nei’s distance, calculated by

$$\begin{array}{c} D=-ln\frac{\sum_{l}\sum_{u}X_{u}Y_{u}}{\sqrt{\left(\sum_{l}\sum_{u}X_{u}^{2}\right)\left(\sum_{l}\sum_{u}Y_{u}^{2}\right)}} \end{array}$$ (1)

was used to create the distance matrix with functions called from the package ‘NAM’ [57]. The built-in R function ‘hclust’ [55] was used to perform an hierarchical cluster analysis using Ward’s minimum-variance method [58], defined by

$$\begin{array}{c} d_{ij}=d\left(\left\{X_{i}\right\},\left\{X_{j}\right\}\right)=\left|\right|X_{i}-X_{j}{\left|\right|}^{2} \end{array}$$ (2)

A genetic clustering diagram, a dendrogram, was created and coded using the package ‘ape’ [59]. The tree was exported in Newick (also known as New Hampshire) file format, then imported into the online application Interactive Tree of Life (iTol) for color annotating [60].

Once the tree was created, the number of sub-groups was determined by a multi-step approach. First, the plots produced from principal component analysis were examined for indications of separation into clear groups. The function ‘cutree’ in RStudio [55] was then used to split the tree into sub-groups based on branch length (genetic dissimilarity), informed generally by the number of clear groups indicated by the principal component plot. Known pedigrees and results of previous studies [25, 36, 47] were then used to identify group names and confirm boundaries. Divisions of between three and eight sub-groups were examined in more detail. Maximum sub-groups were reached when further division did not appear justified based on pedigrees, results of previous studies, as well as the principal component analysis.

### Genetic diversity

To assess the level of genetic variation when dividing the population into three main heterotic groups of SS, NSS, and Iodent, *F*_ST_ was calculated using the package ‘NAM’ [54] in RStudio [55]. This analysis produces estimates of unbiased *F*_ST_ statistics by a weighted analysis of variance method [61]. Overall *F*_ST_ was calculated as the simple average across all loci.

To reduce bias in the *F*_ST_ statistic, two important interrelated modifications were made [62]. Both involved filtering of the inbreds to be used in calculation of the *F*_ST_ statistic. First, to correct for sample size among sub-populations (or heterotic groups, in this case), a balanced number of individuals across the three heterotic groups was selected. Second, to reduce bias of allelic frequencies caused by pedigree structure, the balanced sample from each heterotic group was composed of individuals as genetically unrelated as possible. For example, within the Stiff Stalk heterotic group, the inbreds F42 and B73 are very genetically closely related. Including both of these inbreds in an allelic frequency measure would be essentially using duplicate genotypic data, and would bias the allele frequency calculated for the Stiff Stalk heterotic group. Simply excluding either one inbred, however, while retaining the other, removes the pedigree structure bias while retaining sufficient genetic diversity in the context of *F*_ST_ analysis.

The Iodent heterotic group contained the least number of individuals, so the filtering process was initiated within this subgroup. Filtering of the Iodent subgroup according to the two criteria described above resulted in 44 remaining inbreds. Therefore, in order to balance the data set with equal number of individuals from each heterotic group, 44 became the target number of individuals to select out of the remaining two groups. A list of the inbreds selected for *F*_ST_ analysis is included in the supplemental materials in S3 Table.

For the SNPs with the highest *F*_ST_ values, a candidate gene search was completed for a 10 kbp window on either side of the SNP. This candidate gene search was done within the B73 v2 reference genome, using the R package ‘Zbrowse’ [63].

## Results

### Marker coverage and missing data

As the genotypic data set came from two different sources, it was necessary to merge the genotypic data before analysis. Consequently, out of a total of 955,690 SNP markers in the first set and 546,531 SNP markers in the second set, only 220,550 sites–or 17.2 percent–were common to both GBS sets (Table 3). Following the GBS data set merger and then filtering to remove heterozygous calls, monomorphic sites, markers with greater than 17.2 percent missing data, and SNPs with minor allele frequency (MAF) less than 0.05, the number of SNP markers remaining was 77,314 (Table 4). Missing data may not have been distributed randomly, as use of the B73 reference genome for read alignment causes inbreds closely related to B73 to have a lower proportion of missing data than inbreds more distantly related to B73 [38]. Even so, prior to population analysis, missing data was reduced to zero by imputation. Imputation accuracy was estimated to be 0.83.

### Linkage disequilibrium

**Fig 3:**
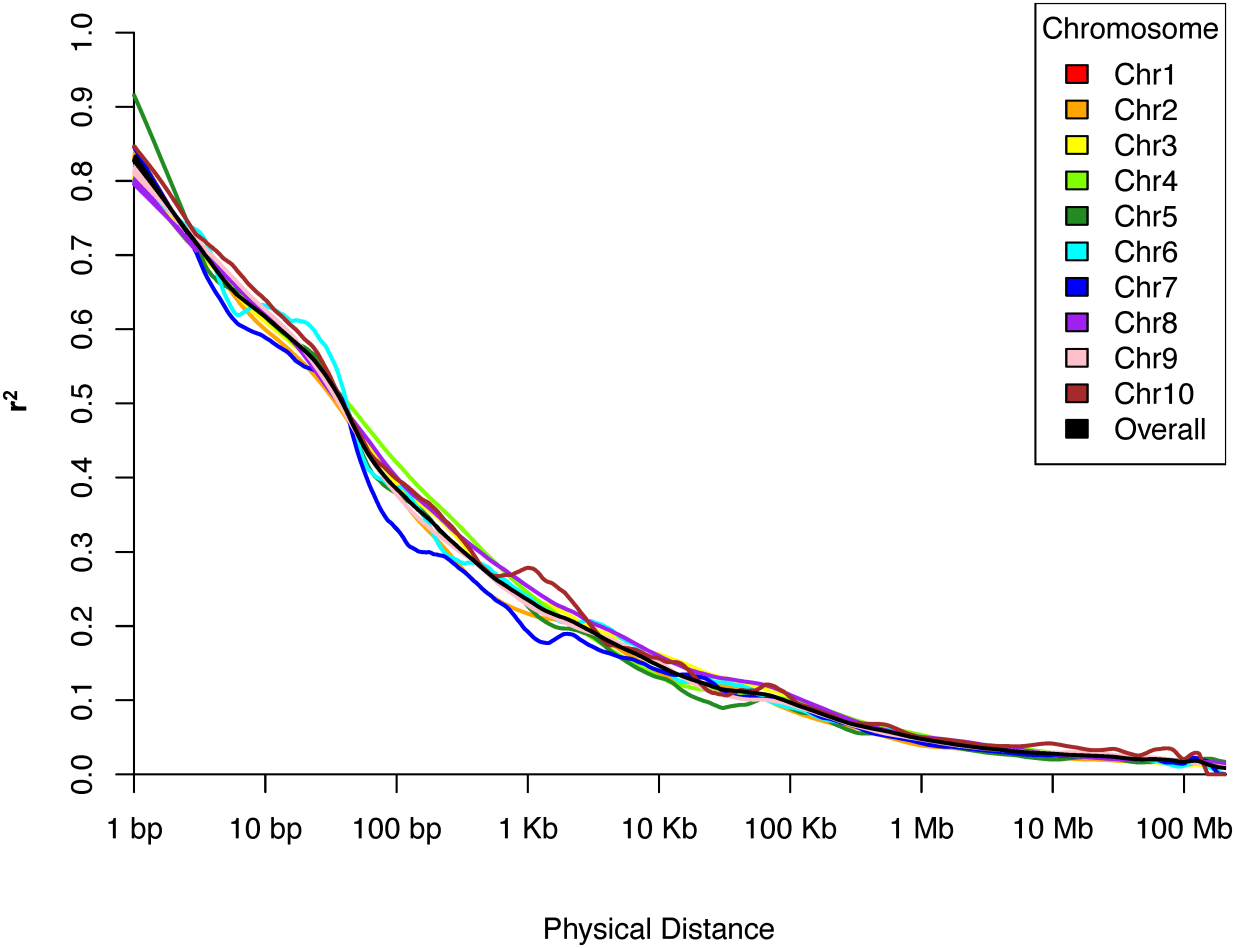
Decay of linkage disequilibrium with physical distance. Decay of linkage disequilibrium (LD) with physical distance between 77,314 pairs of single nucleotide polymorphism (SNP) markers in the ex-PVP and public founder genotypic data set. Physical distance (scaled logarithmically) is on the x-axis and LD, measured in *r*^2^ is on the y-axis. Individual chromosomes are indicated by line color, with the overall average of all data overlaid as a black trend line.

Fig 3 shows the decay of linkage disequilibrium (LD) across genetic distance. An average LD of *r*^2^ at 0.2 was reached at approximately 1.1 Kbp. All chromosomes followed the same general decay trend, with the exception of chromosome 7, which appeared to decay more rapidly than the rest between 100 bp and 1 Kb, reaching an average LD of 0.2 at approximately 1 Kb.

### Population structure

**Fig 4:**
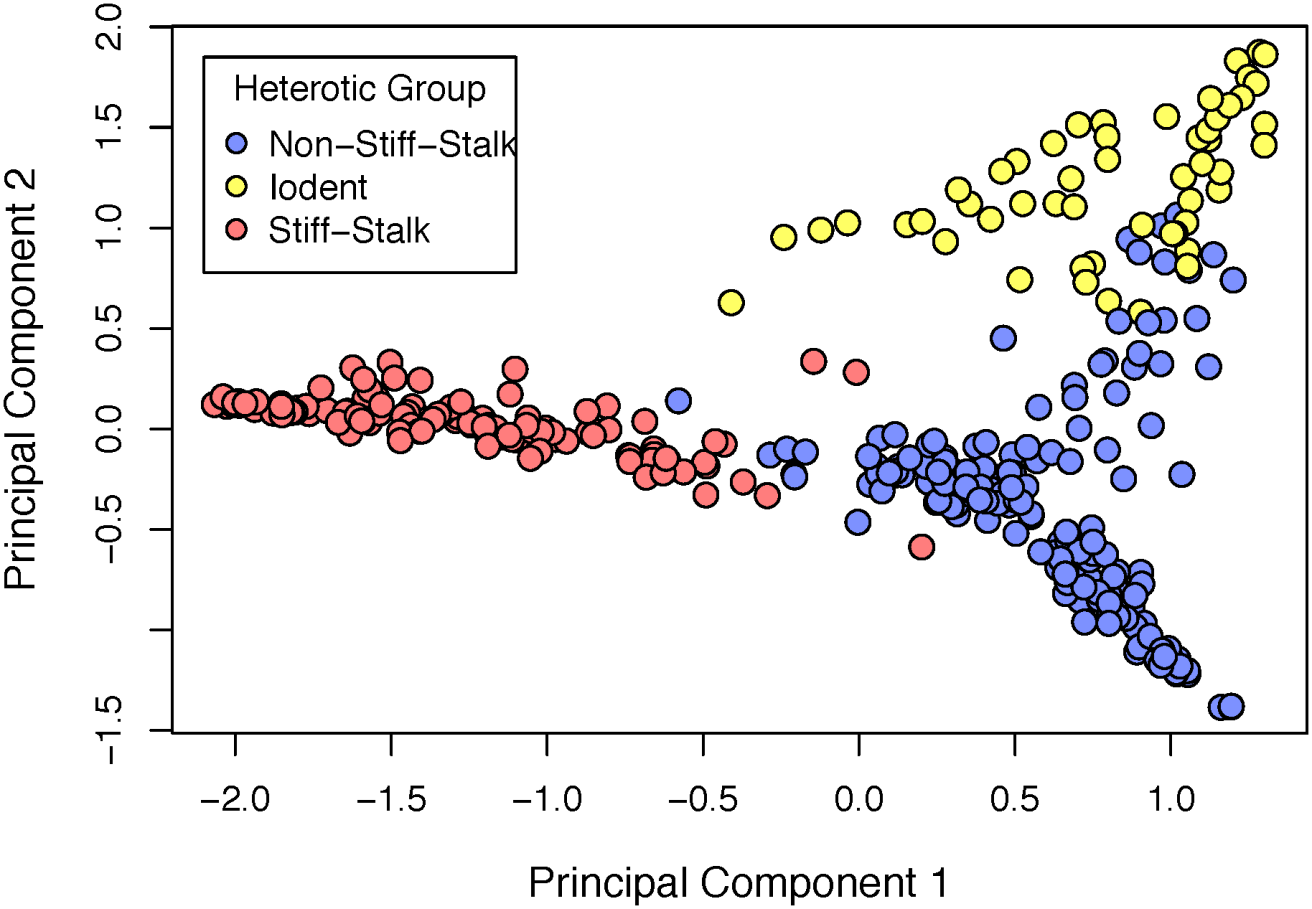
Principal component 1 vs. principal component 2. Principal component no. 1 (x-axis) vs. principal component no. 2 (y-axis), color annotated by three heterotic group divisions. Colors indicate membership in one of three population sub-groups as determined by phylogenetic cluster analysis.

**Fig 5:**
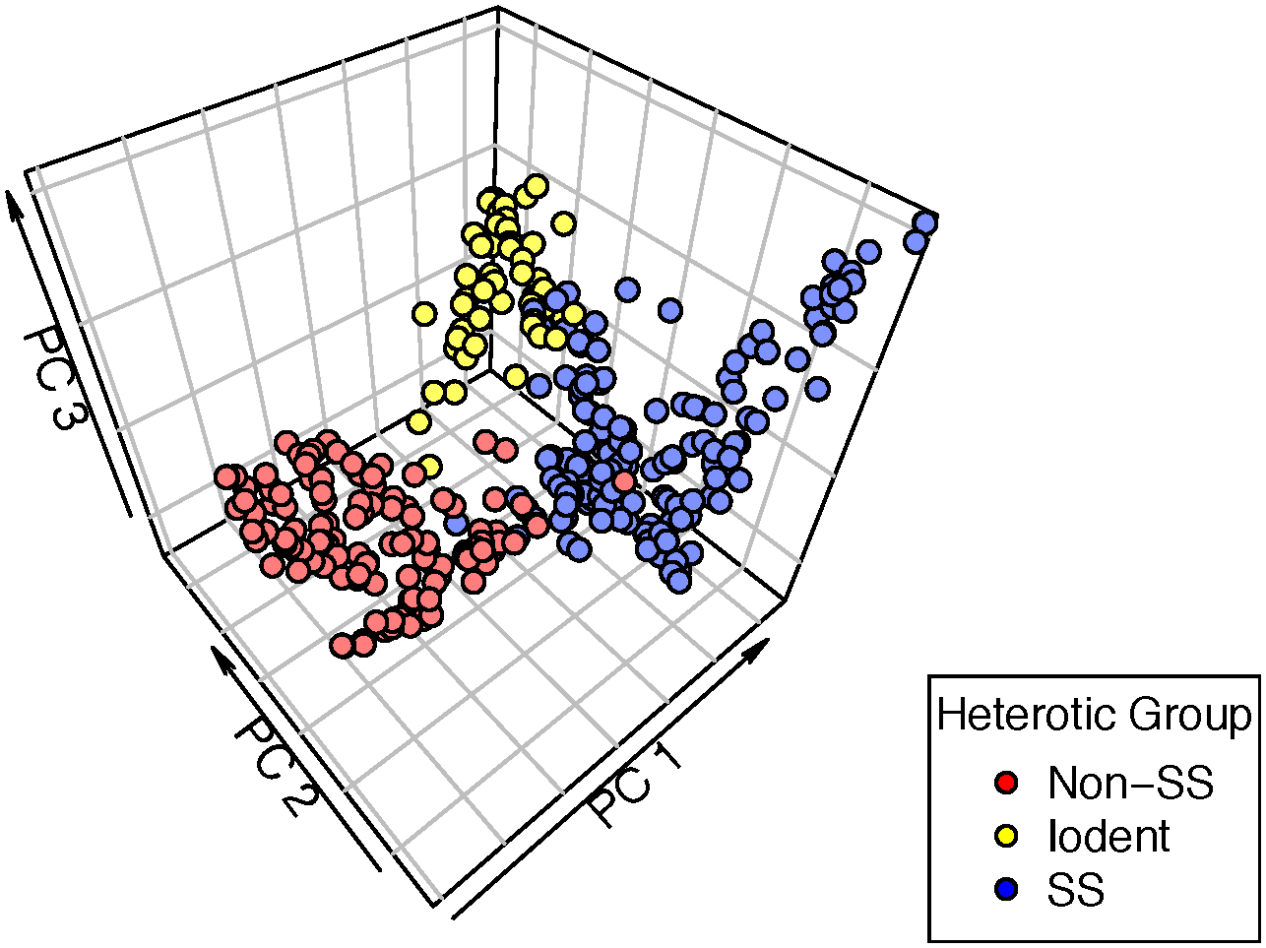
Three-dimensional plot of principal component analysis. Axes labels are abbreviated for principal components 1, 2, and 3, respectively. Colors indicate membership in one of three population sub-groups as determined by phylogenetic cluster analysis.

The ex-PVP inbreds originated from 28 different proprietors (Fig 2 and S1 Table). The public founder inbreds originated from research programs located in 17 different states and one Canadian province (S2 Table). Population stratification was expected to follow the three principal heterotic groups of maize: Stiff Stalk, Non-Stiff Stalk, and Iodent. Two dimensional PC analysis validated this expectation (Fig 4), with three clear spatial divisions in the PCA plot corresponding with the three main population groups identified in the phylogenetic cluster analysis. A PCA plot with three principal components for each inbred line also shows a clear division into three main groups (Fig 5).

**Fig 6:**
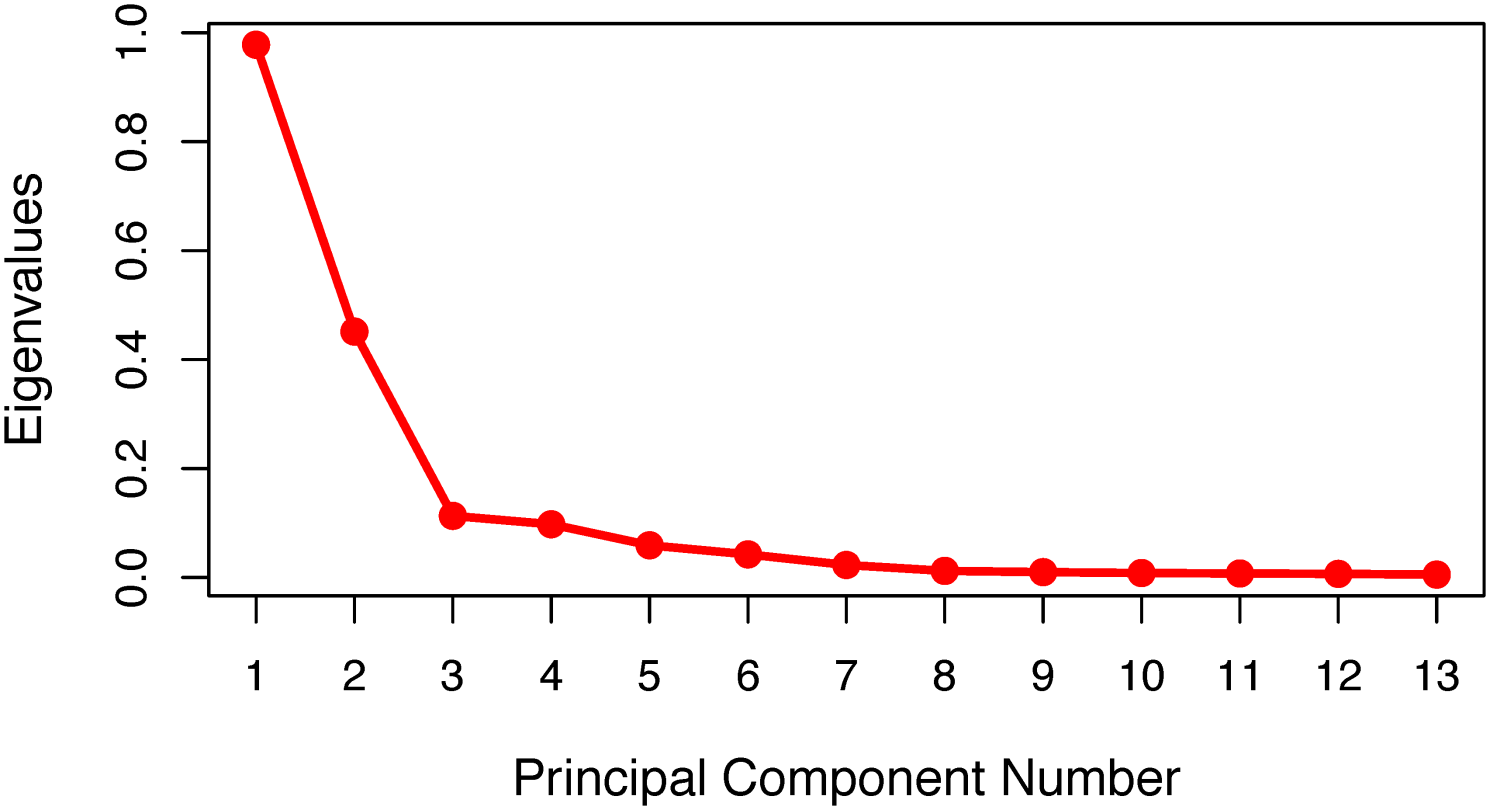
Scree plot of principal component analysis. The number of principal components (PCs) is on the x-axis and the associated eigenvalues–which indicate the amount of variance yet unexplained–are on the y-axis. The optimal number of principal components to explain the variation found in the genotype is found by visually determining the largest point of inflection, or “elbow” of the non-linear trend line [64].

**Fig 7:**
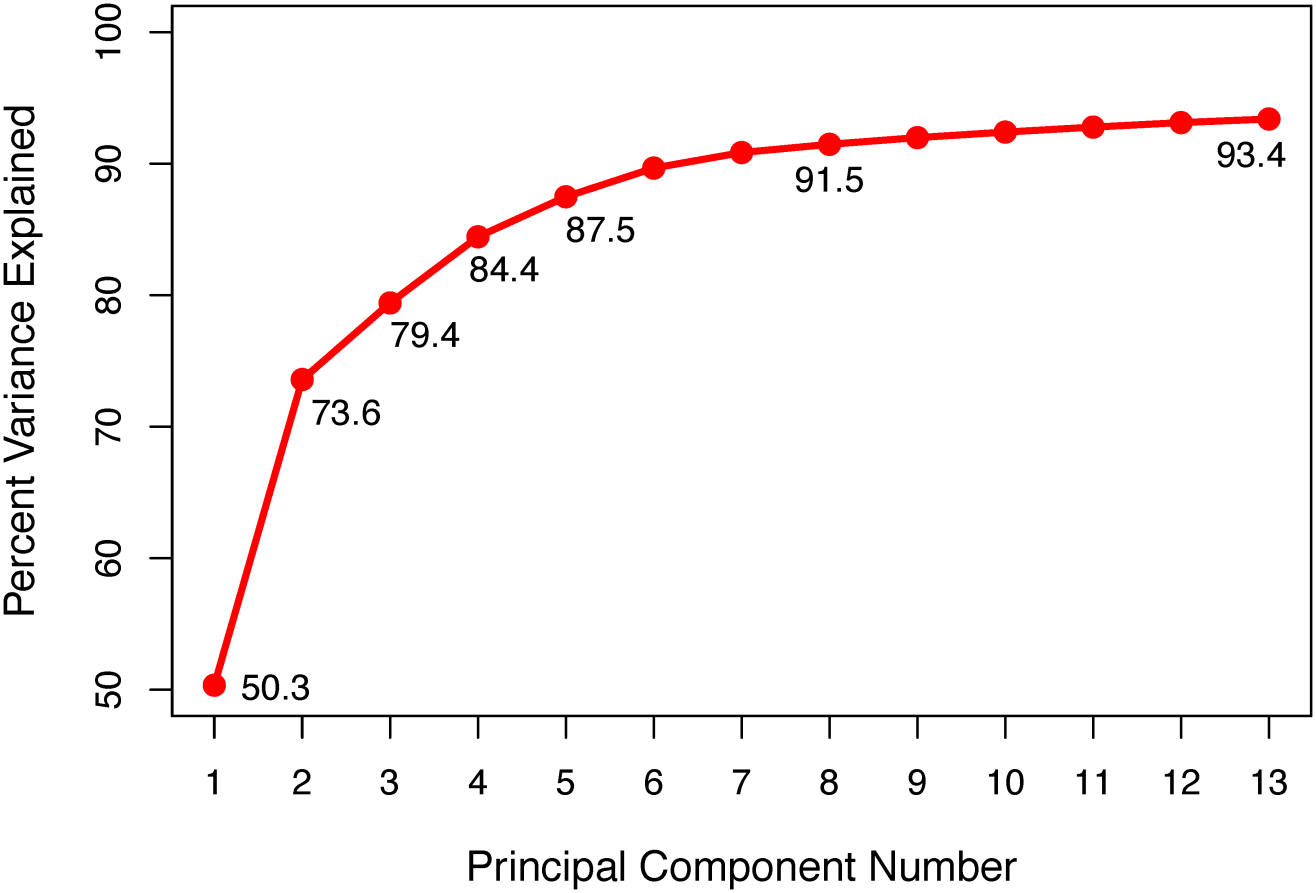
Percent genetic variance explained by principal component analysis. The percent of genetic variance explained is on the y-axis and the principle component (PC) number is on the x-axis. Exact values of percent variance explained are included next to the plotted points at PCs 1-5, 8, and 13.

Further confirmation of the generally expected population stratification is visible in the scree plot of the principal component analysis (Fig 6. The optimal number of principal components to explain genotypic variation, three, was found by visually determining the largest point of inflection, or “elbow” of the non-linear trend line [64]. To find the optimal number of principal components, more complex and empirical methods–such as the silhouette method [65] or the Gap statistic [66]–could have been employed. However, in context of prior knowledge of North American maize heterotic groups as well as phylogenetic cluster analysis based on genotypic data (see next paragraph), the “elbow” method is more than sufficient in this case. Percent variation explained by additional principal components is depicted in Fig 7.

**Fig 8:**
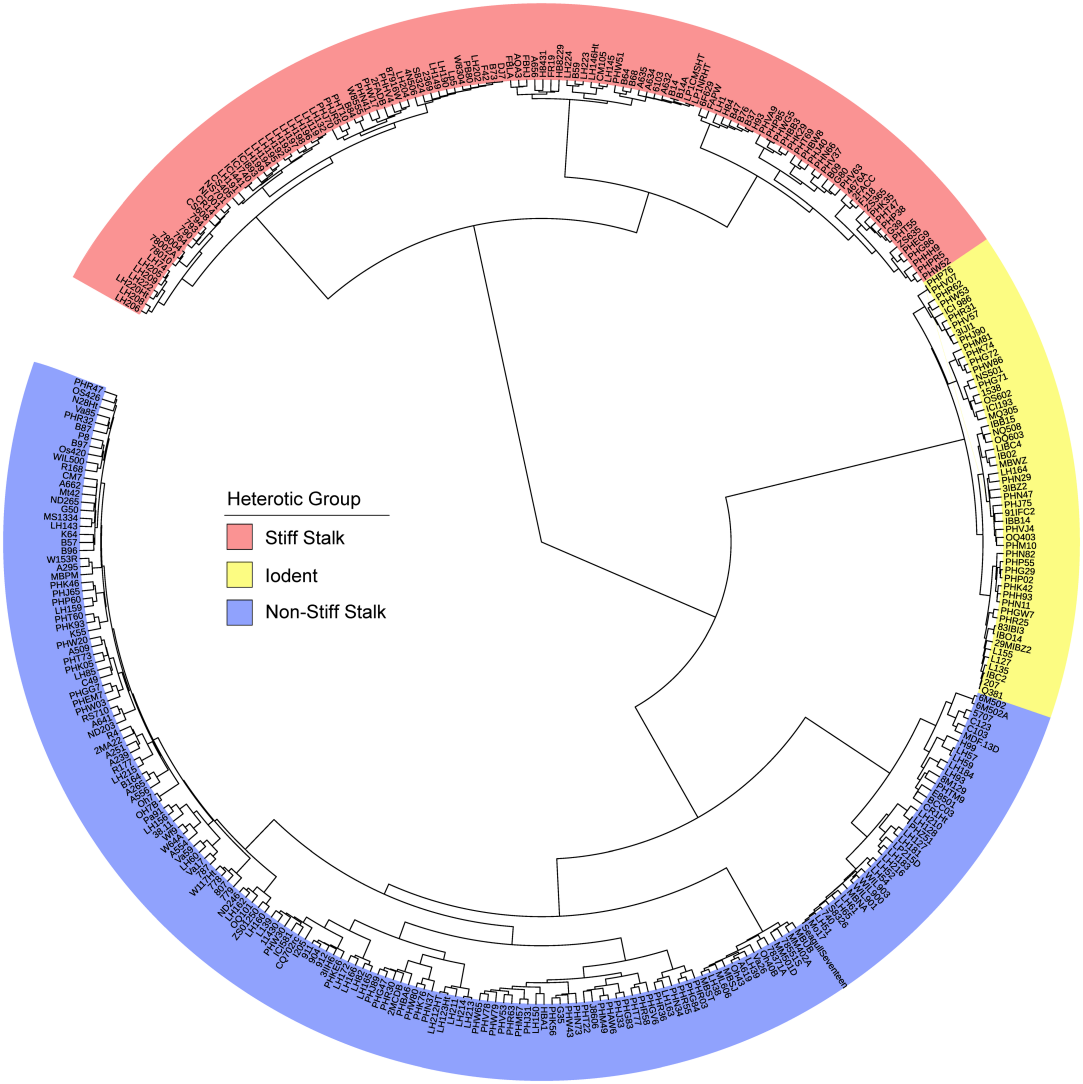
Dendrogram of ex-PVP and public founder inbreds. Circular dendrogram of ex-PVP and public founder inbreds, divided into three heterotic groups. This dendrogram, shown with relative scaled branch lengths and colored according to generally known maize heterotic groups, is based on a cluster analysis using Ward’s minimum distance variance method on the matrix of Nei’s genetic distance [57, 58]. Scaled branch lengths allow a visual representation of the relative proportion of genetic difference between the three main heterotic groups. Consultation of available pedigrees confirm the accuracy of heterotic group placement for individual inbreds [12, 20, 24, 48, 49, 67]. Note: this tree is presented in a rooted format with the primary purpose of illustrating genetic distance while retaining legible inbred names. While no inference is made about common ancestors, the Stiff Stalk and Iodent/Non-Stiff Stalk portions form an ingroup/outgroup interaction, thus ensuring that the presentation of a tree in rooted format is still an acceptable depiction of the detailed population stratification.

Phylogenetic cluster analysis produced a dendrogram that divided into three main groups, Stiff Stalk, Non-Stiff Stalk, and Iodent (Fig 8). General heterotic group assignments based on pedigree data as well as previous publications agree with the classifications assigned by the genotypic clustering method used herein [20, 25, 29, 35, 36, 47]. For a more detailed examination of heterotic group classifications, a dendrogram divided into eight principal population sub-groups was produced by the same methods of cluster analysis. This dendrogram with eight divisions is included in the supplementary materials (see S1 Fig).

### Genetic diversity

**Fig 9:**
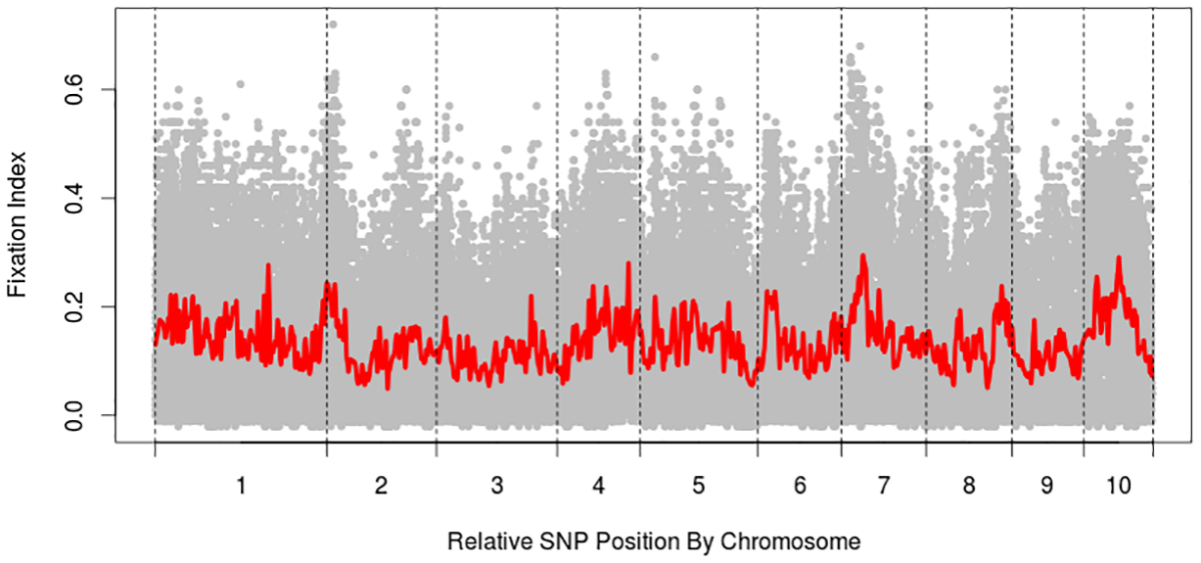
Fixation index values across the genome. Relative SNP position by chromosome (x-axis) and fixation index (y-axis). Each grey dot represents the fixation index statistic (*F*_ST_) for an individual genetic locus. High *F*_ST_ value for a genetic locus may indicate that particular genetic locus contributed to genetic differentiation between heterotic groups. The red trend line represents a moving average across a window of 70 SNPs, or approximately 3570 Mb. This red line is representative, then, of the *F*_ST_ values across genomic regions. Peaks observed in the trend line, particularly in chromosomes 1, 4, 7, and 10, may be indicative of lengthy genomic regions contributing to heterosis observed in hybrid crosses between inbreds from different heterotic groups.

The overall genetic diversity, or mean *F*_ST_, when considering divisions into SS, NSS, and Iodent heterotic groups is 0.1732. Genome-wide *F*_ST_ values plotted against relative marker position are presented in Fig 9. This plot reveals trends that may be worth further study, as they may be indicative of individual loci and/or genomic regions that are involved in population differentiation or possibly even heterosis.

Individual loci were examined for proximity to candidate genes; results are presented in Table 5. These 11 loci are representative of areas where the highest *F*_ST_ values for individual SNPs were found, specifically in chromosomes 2, 4, 5, 7, and 8. The four genomic regions with the highest mean *F*_ST_ value over a window of 70 SNPs are identified in Table 6.

**Table 5:** Candidate genes for SNPs with high *F*_ST_ values.

Chr.	Position^a^	*F*_ST_	Gene ID	Gene Product Description
2	4209922	0.6216	AC210003.2_FG004	Peroxidase 16
			GRMZM2G045049	FGAM synthase
			GRMZM2G342628	-
2	7744913	0.7442	GRMZM2G180870	Glycosyl hydrolases fam. 16 protein
2	11060537	0.6396	GRMZM2G070468	Transferase
			GRMZM2G111232	Protein phosphatase 2C
4	166831567	0.6396	GRMZM2G003501	G14a
5	32460265	0.6686	GRMZM2G139024	Transcription factor Dp-1
7	17142974	0.6657	GRMZM2G019443	AP2 domain cont. protein RAP2.11
7	27658449	0.6537	AC191534.3_FG003	Zinc finger protein
			GRMZM2G162267	-
7	50367063	0.6877	GRMZM2G072868	receptor kinase
7	92941759	0.6023	GRMZM2G129420	Mitochon. ATP synthase subunit
			GRMZM2G129453	Desaturase/cytochrome b5 protein
8	161042132	0.6033	GRMZM2G330719	Cons. gene of unknown function
8	172812044	0.6033	GRMZM2G076410	-

^a^Physical position of the marker within the specified chromosome, in bp.

**Table 6:** Genomic regions with high *F*_ST_ values.

Chr.	First Marker	Last Marker	Region Size	Mean *F*_ST_
1	242575005	245035347	2460342	0.2787
4	166907041	168546722	1639681	0.2885
7	36258886	41462768	5203882	0.3039
10	116450442	117756633	1306191	0.2991

First Marker, Last Marker, and Region Size are all given in bp.

## Discussion

### Linkage disequilibrium

The rate of LD decay reported in maize depends on the population, the genetic region(s) under study, as well as the statistical methods used to compute the values. For the population in this study, the result of LD decay (*r*^2^ at 0.2 at approximately 1.1 Kbp) appears reasonable and well within the general range reported in previous studies. As LD values reported in the literature can vary, the following summary of relevant results of LD decay provides context within which the results of this study can be evaluated.

Developments in genotyping methods led to many studies in the early 2000’s that reported on LD in maize. Tenaillon et al., (2001) [68], reported on a set of 25 maize genotypes comprised of 16 landraces and nine North American inbred lines, analyzing the LD between 21 SNP markers on chromosome 1. Their results showed that LD declines to an average *r*^2^ of 0.2 at 300 bp in the mixed set of 25 genotypes, and greater than 1 kbp in the subset that includes only the nine North American inbreds. Remington et al., (2001) [69] analyzed the LD for 102 inbred lines from a broad range of temperate and tropical origins, using over 1.5 Mb of SSR marker data centered on six candidate gene regions, and found that the decay reached an average of average *r*^2^ of 0.2 at an average of 550 bp for five of the six candidate regions. For the sixth candidate region, LD did not decay to the same level until well after 10 kbp. In another study that examined sequence variants (SNPs and indels) across 18 gene regions in 36 elite U.S. maize inbreds, Ching et al., (2002) [70] concluded that linkage disequilibrium does not significantly decay within the analyzed range of 300-500 bp. Palaisa et al., (2003) [71] found that LD surrounding two loci in a group of 82 diverse inbred lines decayed to a level of *r*^2^ at 0.2 at approximately 1,000 bp. Similarly, in a study of the *adh1* locus within 32 elite North American public and proprietary inbred lines, Jung et al., (2004) [72] reported that while measurable levels of LD appeared to persist past 500 kbp, it could not be stated whether these long-range regions of sustained LD are common. (Estimates of LD at the specific level of *r*^2^ at 0.2 were not available in this study.)

More recently, Yan et al. (2009) [73] studied the extent of LD in 632 maize inbreds from temperate, tropical, and subtropical regions, using 1,229 SNP markers. They found that across all 632 inbreds, LD decayed to an average *r*^2^ of 0.2 at about 500 bp, and generally concluded that the distance of LD decay is much higher in temperate inbreds than in tropical or subtropical inbreds. Truntzler et al., (2012) [74] using a mix of inbreds from public institutions (113 inbreds) and private companies (201 inbreds) and 979 polymorphic SNP markers, reported that while there is a faster rate of LD decay for the private inbreds than the public inbreds, both sets reach *r*^2^ of 0.2 at a distance of about 1-3 kbp. Romay et al. (2013) [38], using a population that is essentially a subset of the population used in this study, found that while the LD for 212 ex-PVP inbreds declined to an average *r*^2^ of 0.2 at 10 kb, the LD decay among public inbreds was much more rapid, reaching *r*^2^ of 0.2 at 1 kb.

The calculation of decay of linkage disequilibrium is affected by many factors: composition of the germplasm set; marker characteristics such as quality and genome coverage; and the analysis method employed. While these factors may lead to variance between data sets of the physical distance observed at the standard-reported linkage equilibrium value *r*^2^ of 0.2, the general trends found in this germplasm set are consistent with the results previously reported.

### Population structure

Inclusion of a scale in the dendrogram (Fig 8) allows inferences to be made about relative genetic distance between heterotic groups. A comparison between the three heterotic groups in Fig 8 reinforces the concept that commercial breeding efforts in the Plant Variety Protection era (post-1970) have continued to drive genetic divergence among Stiff Stalk, Non-Stiff Stalk, and Iodent. *F*_ST_ analysis confirms the divergence, with the result of 0.1732 indicating moderate levels of divergence among these three heterotic groups. A more detailed look at the *F*_ST_ analysis reveals several genomic regions as well as several individual SNPs with high *F*_ST_ values. High *F*_ST_ values for a particular region or SNP mean that it is more likely that the major haplotype or allele in the SS group is different than the major haplotype or allele in the NSS group. These genomic regions and individual SNPs that show high genetic diversity between heterotic groups deserve further study, as they may provide insights about the genetic basis of heterosis.

It is widely accepted among breeders and others familiar with North American maize germplasm that heterotic groups continue to diverge genetically [36, 75]. One reason for this genetic divergence of heterotic groups could be the widespread breeding practice of recycling of elite inbreds within heterotic groups to produce new inbreds, then evaluating them based on testcross performance with inbreds from other heterotic groups. The observed genetic divergence between breeding pools then may be a response to selection for heterosis in testcross hybrids. The value of a commercial inbred is not just based on its ability to efficiently produce hybrid seed, but primarily on its ability to consistently produce superior grain yield in a testcross. Therefore, as inbreds are judged by their performance in a testcross, a higher degree of genetic divergence between heterotic groups may be a result of selection over time for better hybrid performance.

### Application of genetic relationships in breeding

Precise and accurate knowledge of the genetic background of a particular inbred can be very useful to a plant breeder in determining the best use of that inbred. Traditional pedigree information, supplemented by population genetics data can help a breeder decide what combination of inbreds may prove to be the best for breeding crosses and for hybrid testcrosses. Many PVP inbreds came from self-pollination of commercial hybrids. An accurate dendrogram based on genetic relationships can help breeders better understand the genetic background of PVP inbreds derived from commercial hybrids, as well as identify close genetic relatives. For one example, the P3737-derived inbreds 3IIH6, 912, 904, and 911 are located near the bottom of the tree in S9 Fig. The location of these lines within the dendrogram does not align with expectations based only on field testing and general pedigree knowledge. With robust genetic diversity analysis, however, a more clear and complete picture emerges.

The dendrogram produced in this study (Fig 8) visually identifies the heterotic group membership of each ex-PVP and pubic inbred. The divisions among Stiff Stalk, Non-Stiff Stalk, and Iodent are clear. Further sub-group divisions within the Stiff Stalk heterotic group are defined. However, the sub-group divisions within the Non-Stiff Stalk heterotic group are more difficult to resolve (see S1 Fig). Many of the ex-PVP Non-Stiff Stalk inbreds are genetically closely related, especially in the “Pioneer Mixed” and “Miscellaneous” groups. This study includes a larger number of ex-PVP inbreds and more detailed information about relationships derived directly from genotypic cluster analysis than previous studies. In general, the results presented here agree with previous classifications of maize heterotic groups [21, 25, 36, 47].

Information from this study can be useful in determining how to begin testing a newly released ex-PVP inbred line. When the PVP certificate for an inbred expires and the seed is freely available for use, the parentage of the line can be determined by consulting the pedigree on the certificate. Then the parental inbreds can be located on the dendrogram. Thus, the newly released ex-PVP inbred can be anchored to previously characterized inbreds. Such an approach can potentially save time and resources, particularly for smaller breeding programs.

Previous yield trial results of parental lines could be a logical starting point for determining the potential combining patterns and agronomic performance value of a newly released ex-PVP inbred [76]. Alternatively, if the pedigree on the certificate does not include parental inbreds that are within the current genetic cluster diagram, and if the inbred can be quickly genotyped, then the inbred can be included in a new cluster analysis where the precise genetic relationships can be determined. Even if good parental pedigree and testcross data is available for a newly expired PVP inbred, there may be merit to genotyping the inbred and determining where it falls in the cluster diagram, as this provides complementary and more precise genetic relationship information. Yield trial data coupled with this population genetic analysis may further improve a breeder’s ability to immediately identify the best material and quickly integrate it into a germplasm pool. Understanding the genetic relationships and population differentiation of elite maize germplasm is an integral part part of helping breeders to maintain and potentially increase the rate of genetic gain, resulting in higher overall agronomic performance of inbreds and hybrids.

## Supporting information

S1 Table Ex-PVP inbreds used in this study. (CSV)

S2 Table Public inbreds used in this study. (CSV)

S3 Table Subsets of inbreds used in *F_ST_* calculations. (CSV)

S4 Table List of individual accession identifiers for all inbreds used in this study for genotyping-by-sequencing (GBS) data, available at www.panzea.org [50]. In the data repository, individual accession identifiers are referred to as “Taxa”, and thus have been listed as such in this table.
(CSV)

S1 Fig Dendrogram of ex-PVP and public founder inbreds, divided into eight heterotic groups. Shown with relative scaled branch lengths, this dendrogram is based on a cluster analysis using Ward’s minimum distance variance method, and Nei’s genetic distance [57, 58]. Colors represent further divisions of heterotic groups of maize, with groups named by important founder line or by general group composition. Consultation of published pedigrees [48, 49, 67] as well as previous publications on the subject as well as previous publications on the subject [12, 20, 24] confirm the accuracy of heterotic group placement for individual inbreds.
(TIFF)

S2 Fig Linear dendrogram of ex-PVP and public founder inbreds, divided into eight sub-groups. This dendrogram is based on phylogenetic cluster analysis using Ward’s minimum distance variance method, and Nei’s genetic distance [57, 58]. Tree branch lengths are scaled relatively according to the actual genetic distance matrix. Colors correlate with maize family groups as indicated in the “Heterotic Group” key. Pedigrees are included to the right of each inbred. PVP inbred pedigrees were obtained from from PVP certificates, available at ars.grin.gov [48]. Public inbred pedigrees were obtained from Gerdes et al., (1993) [49] and Cross et al., (1989) [67]. Consultation of pedigrees, as well as previous publications on the subject [12, 20, 24], confirm individual heterotic group memberships are accurate.
(TIFF)

S3 Fig B73 group dendrogram with inbred pedigrees. The color surrounding the ex-PVP and public inbred names corresponds with the color assigned to each family subgroup in S1 and S2 Figs. Pedigrees are included to the right of each inbred. PVP inbred pedigrees were obtained from from PVP certificates, available at ars.grin.gov [48]. Public inbred pedigrees were obtained from Gerdes et al., (1993) [49] and Cross et al., (1989) [67]. Consultation of pedigrees, as well as previous publications on the subject [12, 20, 24], confirm individual heterotic group memberships are accurate.
(TIFF)

S4 Fig B14 group dendrogram with inbred pedigrees. The color surrounding the ex-PVP and public inbred names corresponds with the color assigned to each family subgroup in S1 and S2 Figs. Pedigrees are included to the right of each inbred. PVP inbred pedigrees were obtained from from PVP certificates, available at ars.grin.gov [48]. Public inbred pedigrees were obtained from Gerdes et al., (1993) [49] and Cross et al., (1989) [67]. Consultation of pedigrees, as well as previous publications on the subject [12, 20, 24], confirm individual heterotic group memberships are accurate.
(TIFF)

S5 Fig B37 group dendrogram with inbred pedigrees. The color surrounding the ex-PVP and public inbred names corresponds with the color assigned to each family subgroup in S1 and S2 Figs. Pedigrees are included to the right of each inbred. PVP inbred pedigrees were obtained from from PVP certificates, available at ars.grin.gov [48]. Public inbred pedigrees were obtained from Gerdes et al., (1993) [49] and Cross et al., (1989) [67]. Consultation of pedigrees, as well as previous publications on the subject [12, 20, 24], confirm individual heterotic group memberships are accurate.
(TIFF)

S6 Fig Iodent group dendrogram with inbred pedigrees. The color surrounding the ex-PVP and public inbred names corresponds with the color assigned to each family subgroup in S1 and S2 Figs. Pedigrees are included to the right of each inbred. PVP inbred pedigrees were obtained from from PVP certificates, available at ars.grin.gov [48]. Public inbred pedigrees were obtained from Gerdes et al., (1993) [49] and Cross et al., (1989) [67]. Consultation of pedigrees, as well as previous publications on the subject [12, 20, 24], confirm individual heterotic group memberships are accurate.
(TIFF)

S7 Fig Lancaster group dendrogram with inbred pedigrees. The color surrounding the ex-PVP and public inbred names corresponds with the color assigned to each family subgroup in S1 and S2 Figs. Pedigrees are included to the right of each inbred. PVP inbred pedigrees were obtained from from PVP certificates, available at ars.grin.gov [48]. Public inbred pedigrees were obtained from Gerdes et al., (1993) [49] and Cross et al., (1989) [67]. Consultation of pedigrees, as well as previous publications on the subject [12, 20, 24], confirm individual heterotic group memberships are accurate.
(TIFF)

S8 Fig Ohio 43 group dendrogram with inbred pedigrees. The color surrounding the ex-PVP and public inbred names corresponds with the color assigned to each family subgroup in S1 and S2 Figs. Pedigrees are included to the right of each inbred. PVP inbred pedigrees were obtained from from PVP certificates, available at ars.grin.gov [48]. Public inbred pedigrees were obtained from Gerdes et al., (1993) [49] and Cross et al., (1989) [67]. Consultation of pedigrees, as well as previous publications on the subject [12, 20, 24], confirm individual heterotic group memberships are accurate.
(TIFF)

S9 Fig Pioneer Mixed group dendrogram with inbred pedigrees. The color surrounding the ex-PVP and public inbred names corresponds with the color assigned to each family subgroup in S1 and S2 Figs. Pedigrees are included to the right of each inbred. PVP inbred pedigrees were obtained from from PVP certificates, available at ars.grin.gov [48]. Public inbred pedigrees were obtained from Gerdes et al., (1993) [49] and Cross et al., (1989) [67]. Consultation of pedigrees, as well as previous publications on the subject [12, 20, 24], confirm individual heterotic group memberships are accurate.
(TIFF)

S10 Fig Miscellaneous sub-group dendrogram with pedigrees. The color surrounding the ex-PVP and public inbred names corresponds with the color assigned to each family subgroup in S1 and S2 Figs. Pedigrees are included to the right of each inbred. PVP inbred pedigrees were obtained from from PVP certificates, available at ars.grin.gov [48]. Public inbred pedigrees were obtained from Gerdes et al., (1993) [49] and Cross et al., (1989) [67]. Consultation of pedigrees, as well as previous publications on the subject [12, 20, 24], confirm individual heterotic group memberships are accurate.
(TIFF)
