# Supplementary figures and images for: Genetic relatedness of previously Plant-Variety-Protected commercial maize inbreds
Source: PLoS One. 2017 Dec 13;12(12):e0189277. doi: 10.1371/journal.pone.0189277 (PMC5728570; doi:10.1371/journal.pone.0189277)

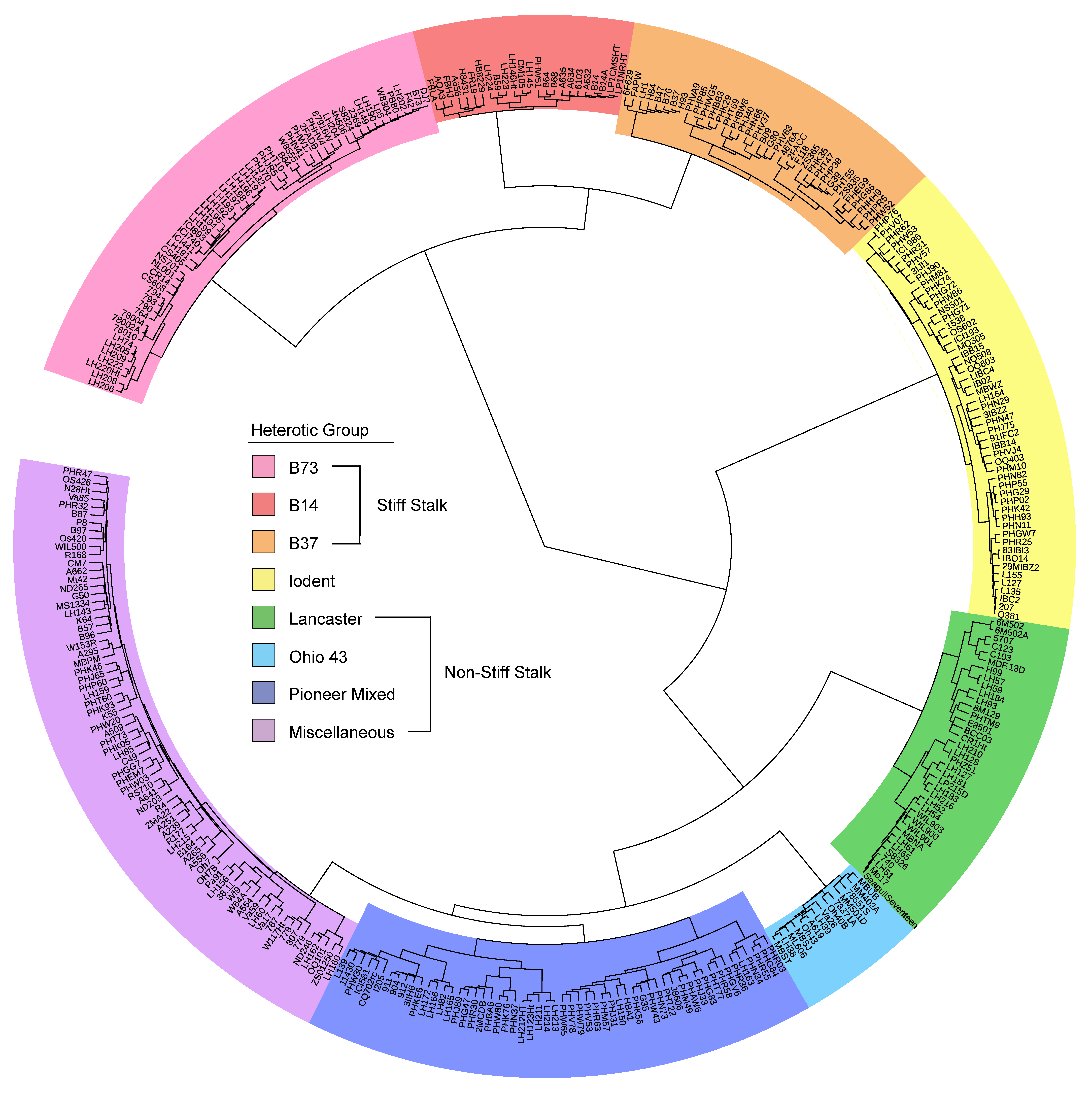

Supplement: S1 Fig — Shown with relative scaled branch lengths, this dendrogram is based on a cluster analysis using Ward’s minimum distance variance method, and Nei’s genetic distance [57, 58]. Colors represent further divisions of heterotic groups of maize, with groups named by important founder line or by general group composition. Consultation of published pedigrees [48, 49, 67] as well as previous publications on the subject as well as previous publications on the subject [12, 20, 24] confirm the accuracy of heterotic group placement for individual inbreds. (TIFF) [file pone.0189277.s005.tiff]

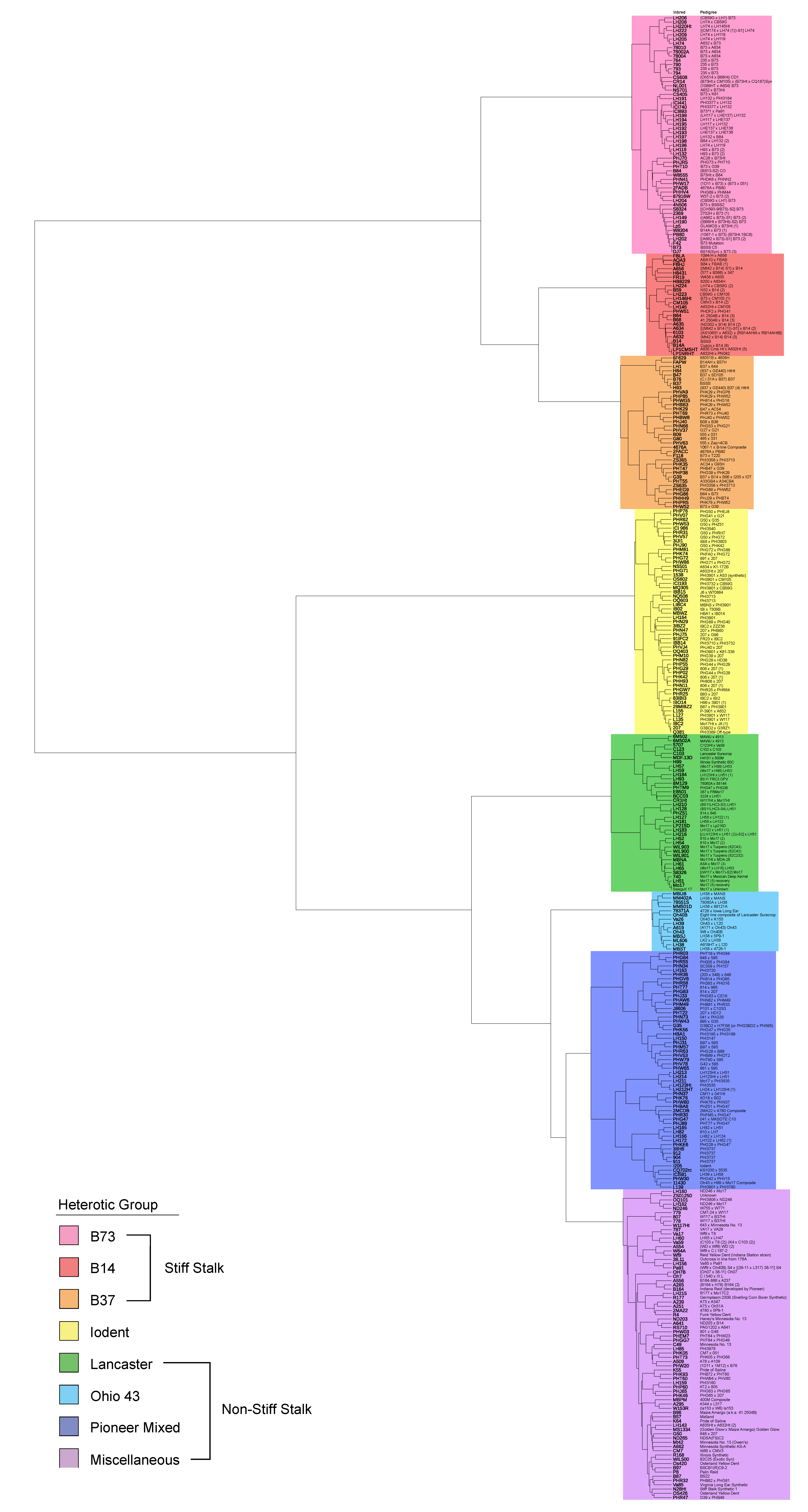

Supplement: S2 Fig — This dendrogram is based on phylogenetic cluster analysis using Ward’s minimum distance variance method, and Nei’s genetic distance [57, 58]. Tree branch lengths are scaled relatively according to the actual genetic distance matrix. Colors correlate with maize family groups as indicated in the “Heterotic Group” key. Pedigrees are included to the right of each inbred. PVP inbred pedigrees were obtained from from PVP certificates, available at ars.grin.gov [48]. Public inbred pedigrees were obtained from Gerdes et al., (1993) [49] and Cross et al., (1989) [67]. Consultation of pedigrees, as well as previous publications on the subject [12, 20, 24], confirm individual heterotic group memberships are accurate. (TIFF) [file pone.0189277.s006.tiff]

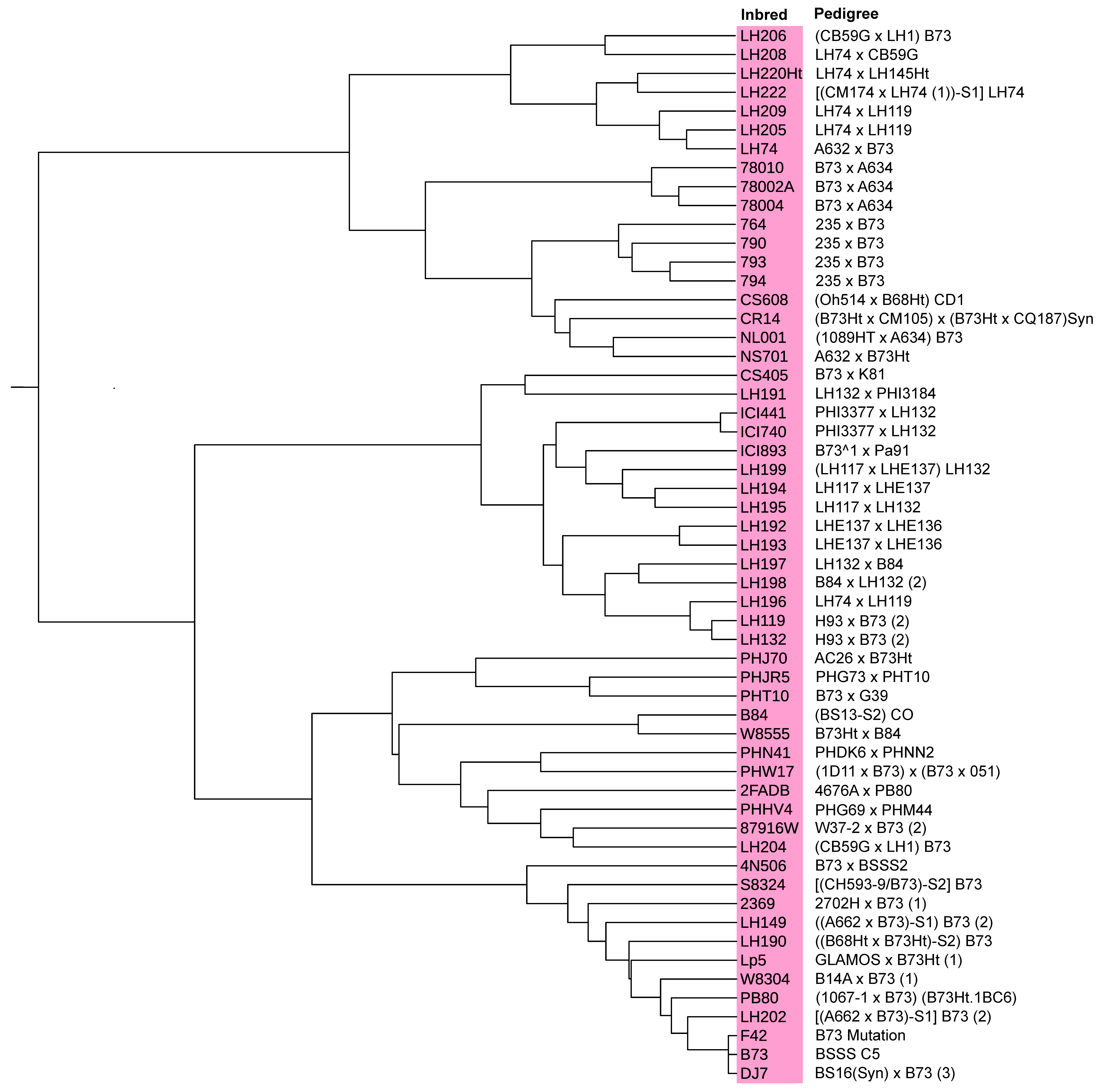

Supplement: S3 Fig — The color surrounding the ex-PVP and public inbred names corresponds with the color assigned to each family subgroup in S1 and S2 Figs. Pedigrees are included to the right of each inbred. PVP inbred pedigrees were obtained from from PVP certificates, available at ars.grin.gov [48]. Public inbred pedigrees were obtained from Gerdes et al., (1993) [49] and Cross et al., (1989) [67]. Consultation of pedigrees, as well as previous publications on the subject [12, 20, 24], confirm individual heterotic group memberships are accurate. (TIFF) [file pone.0189277.s007.tiff]

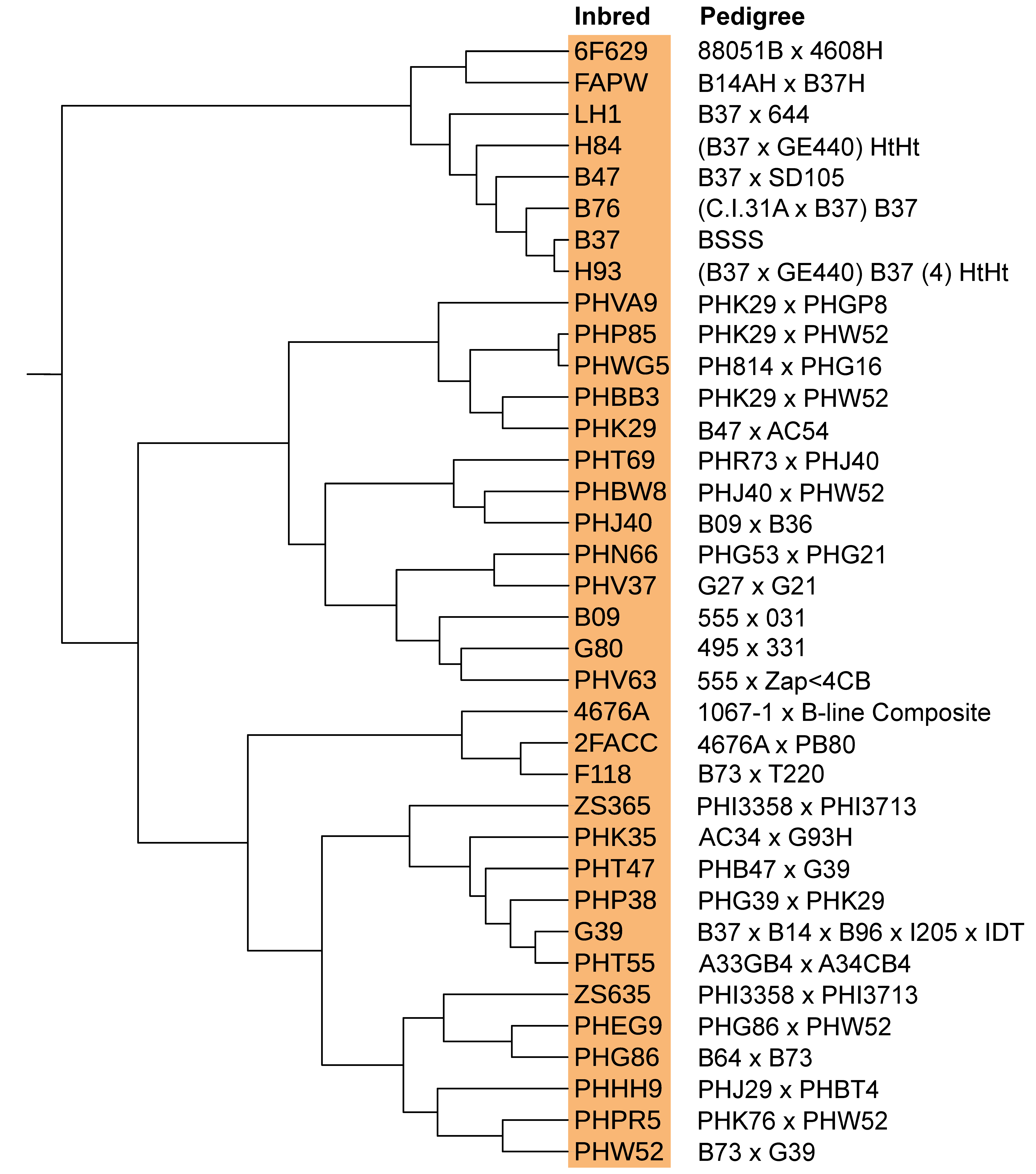

Supplement: S5 Fig — The color surrounding the ex-PVP and public inbred names corresponds with the color assigned to each family subgroup in S1 and S2 Figs. Pedigrees are included to the right of each inbred. PVP inbred pedigrees were obtained from from PVP certificates, available at ars.grin.gov [48]. Public inbred pedigrees were obtained from Gerdes et al., (1993) [49] and Cross et al., (1989) [67]. Consultation of pedigrees, as well as previous publications on the subject [12, 20, 24], confirm individual heterotic group memberships are accurate. (TIFF) [file pone.0189277.s009.tiff]

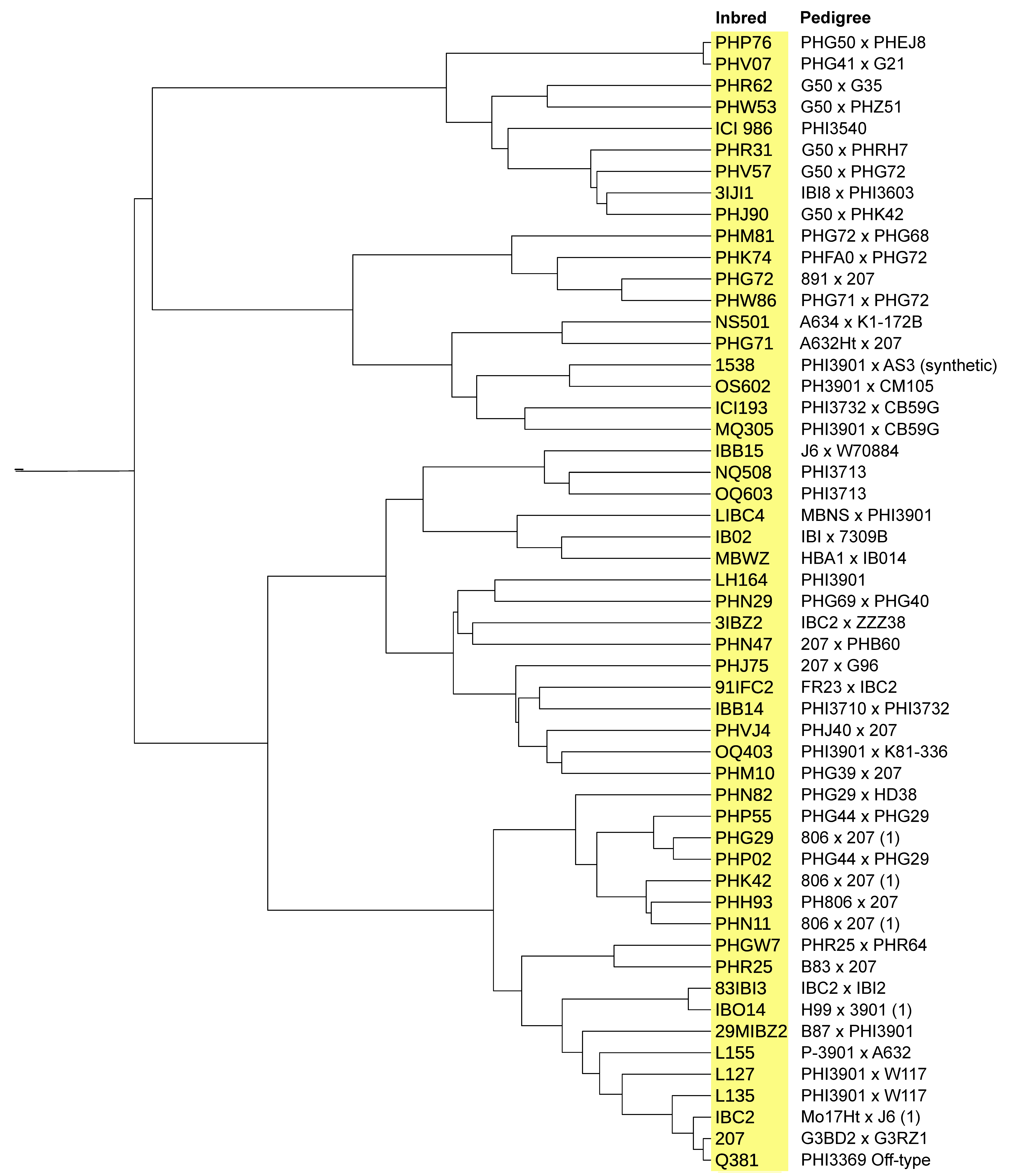

Supplement: S6 Fig — The color surrounding the ex-PVP and public inbred names corresponds with the color assigned to each family subgroup in S1 and S2 Figs. Pedigrees are included to the right of each inbred. PVP inbred pedigrees were obtained from from PVP certificates, available at ars.grin.gov [48]. Public inbred pedigrees were obtained from Gerdes et al., (1993) [49] and Cross et al., (1989) [67]. Consultation of pedigrees, as well as previous publications on the subject [12, 20, 24], confirm individual heterotic group memberships are accurate. (TIFF) [file pone.0189277.s010.tiff]

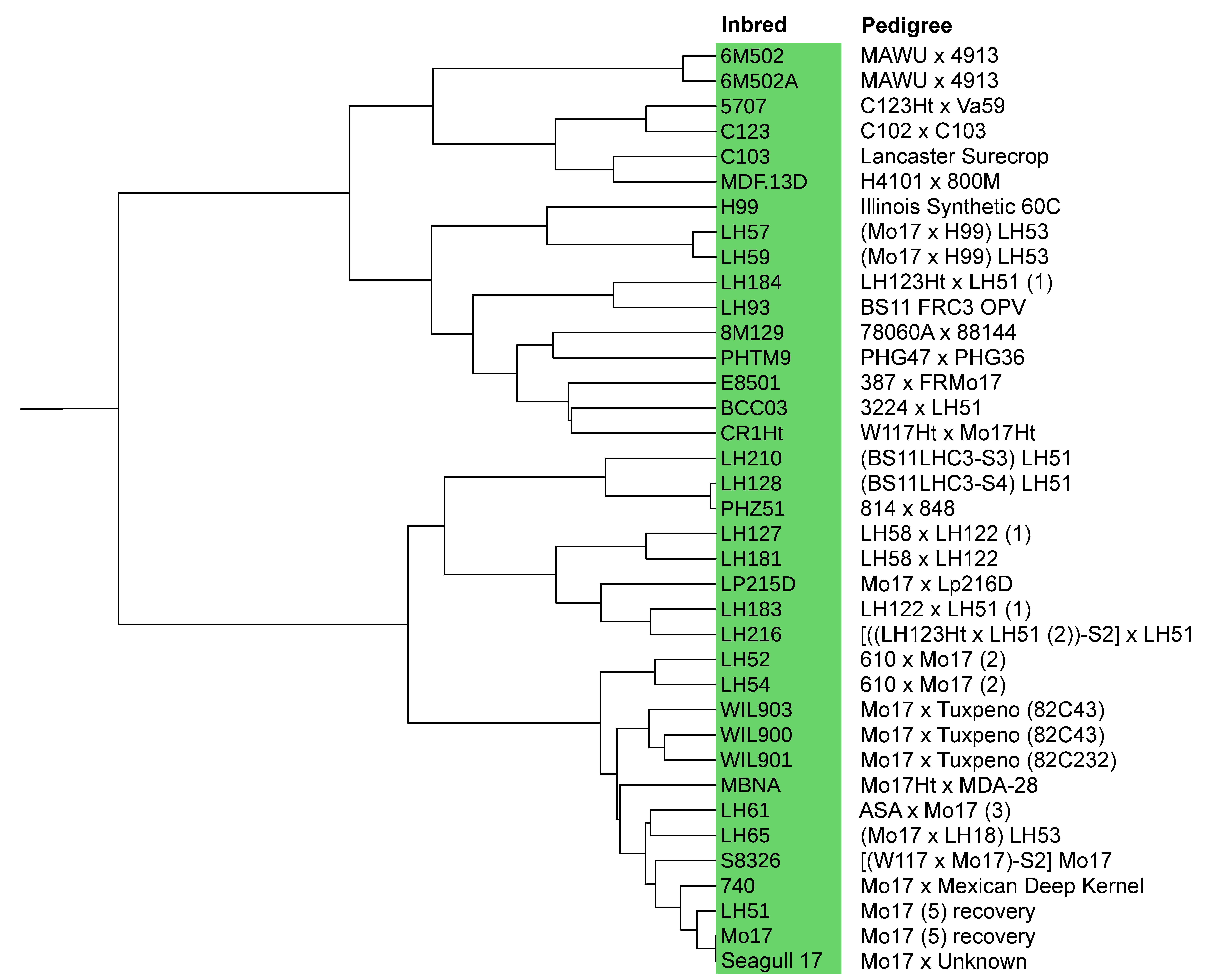

Supplement: S7 Fig — The color surrounding the ex-PVP and public inbred names corresponds with the color assigned to each family subgroup in S1 and S2 Figs. Pedigrees are included to the right of each inbred. PVP inbred pedigrees were obtained from from PVP certificates, available at ars.grin.gov [48]. Public inbred pedigrees were obtained from Gerdes et al., (1993) [49] and Cross et al., (1989) [67]. Consultation of pedigrees, as well as previous publications on the subject [12, 20, 24], confirm individual heterotic group memberships are accurate. (TIFF) [file pone.0189277.s011.tiff]

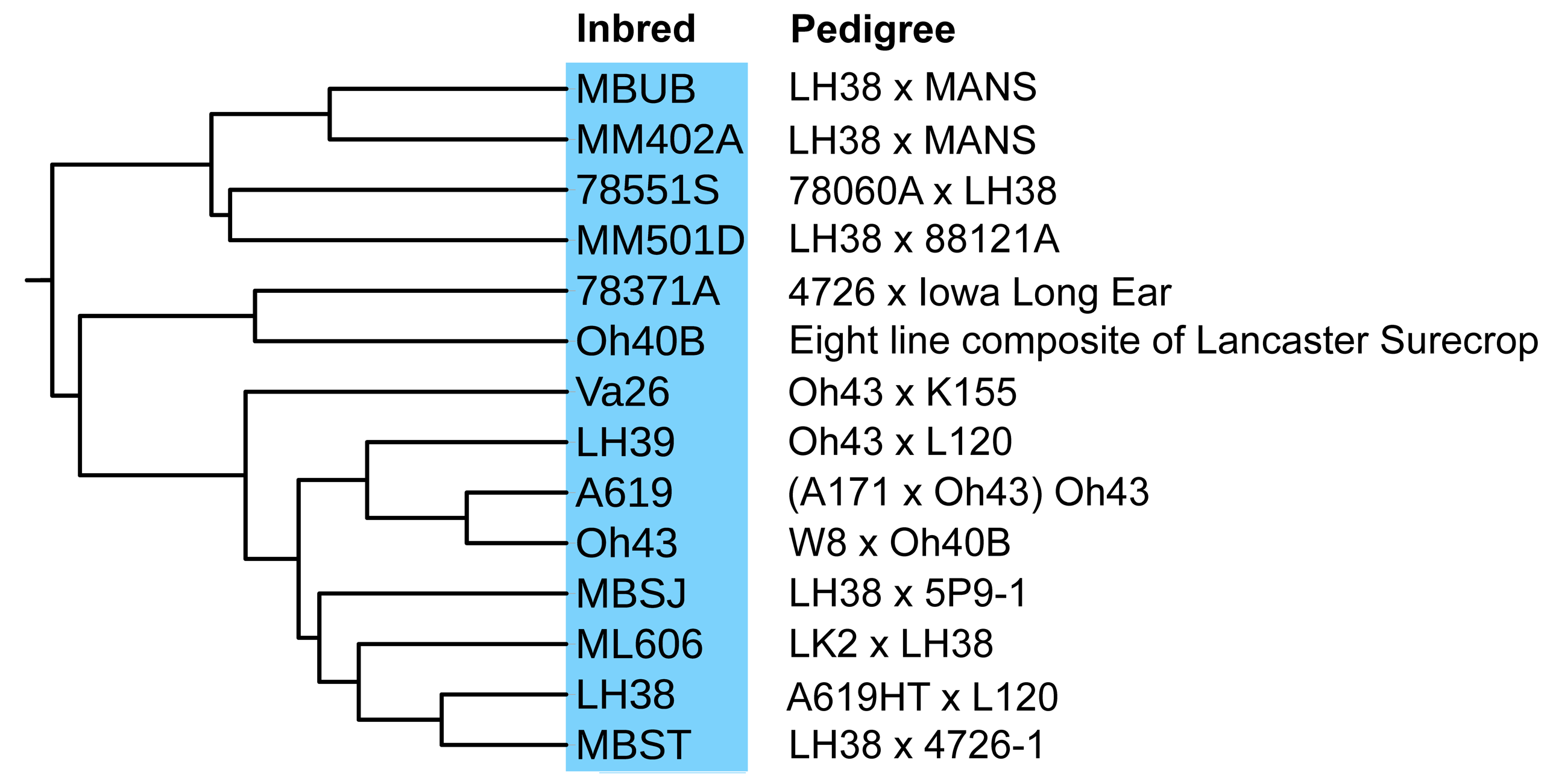

Supplement: S8 Fig — The color surrounding the ex-PVP and public inbred names corresponds with the color assigned to each family subgroup in S1 and S2 Figs. Pedigrees are included to the right of each inbred. PVP inbred pedigrees were obtained from from PVP certificates, available at ars.grin.gov [48]. Public inbred pedigrees were obtained from Gerdes et al., (1993) [49] and Cross et al., (1989) [67]. Consultation of pedigrees, as well as previous publications on the subject [12, 20, 24], confirm individual heterotic group memberships are accurate. (TIFF) [file pone.0189277.s012.tiff]

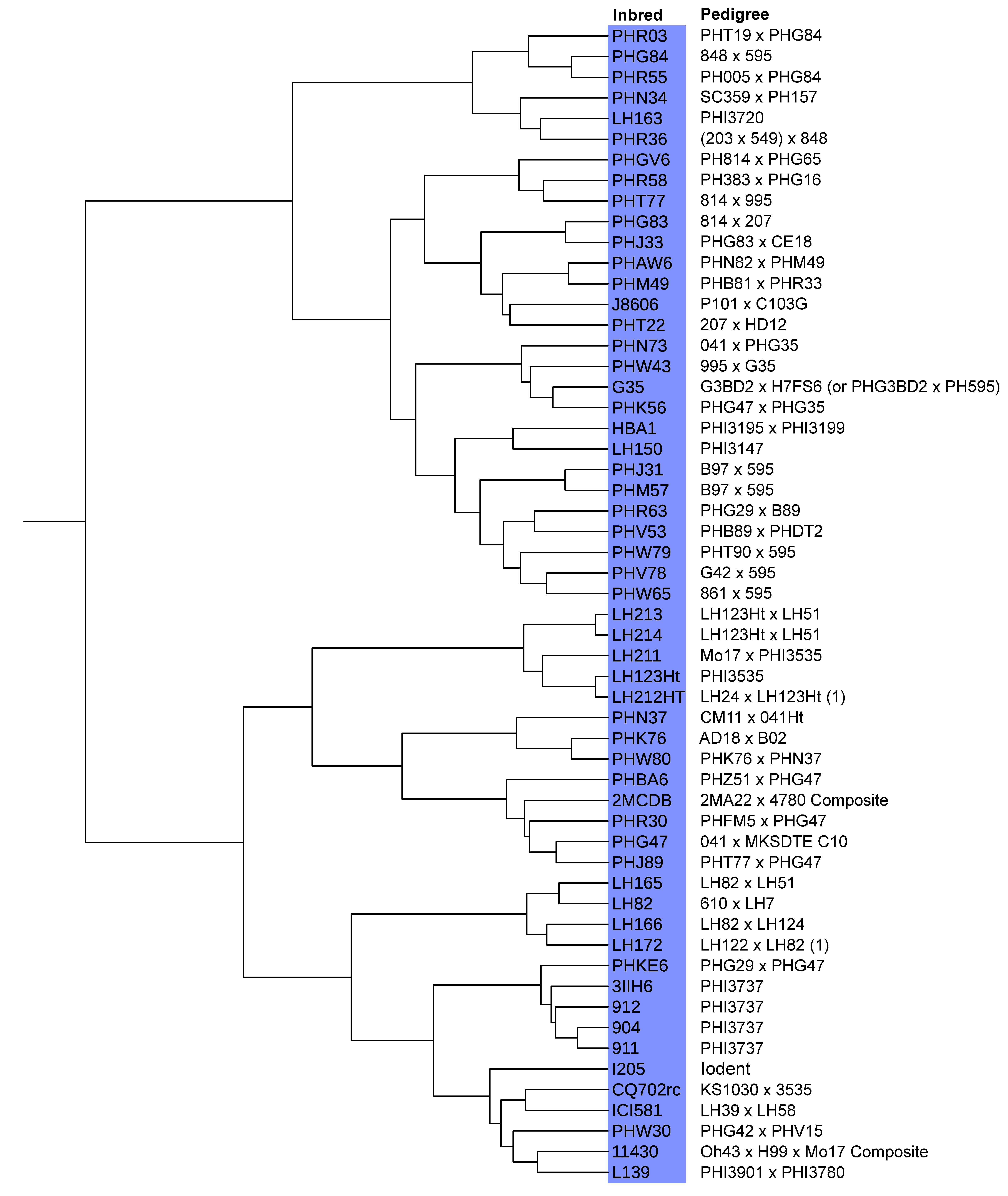

Supplement: S9 Fig — The color surrounding the ex-PVP and public inbred names corresponds with the color assigned to each family subgroup in S1 and S2 Figs. Pedigrees are included to the right of each inbred. PVP inbred pedigrees were obtained from from PVP certificates, available at ars.grin.gov [48]. Public inbred pedigrees were obtained from Gerdes et al., (1993) [49] and Cross et al., (1989) [67]. Consultation of pedigrees, as well as previous publications on the subject [12, 20, 24], confirm individual heterotic group memberships are accurate. (TIFF) [file pone.0189277.s013.tiff]

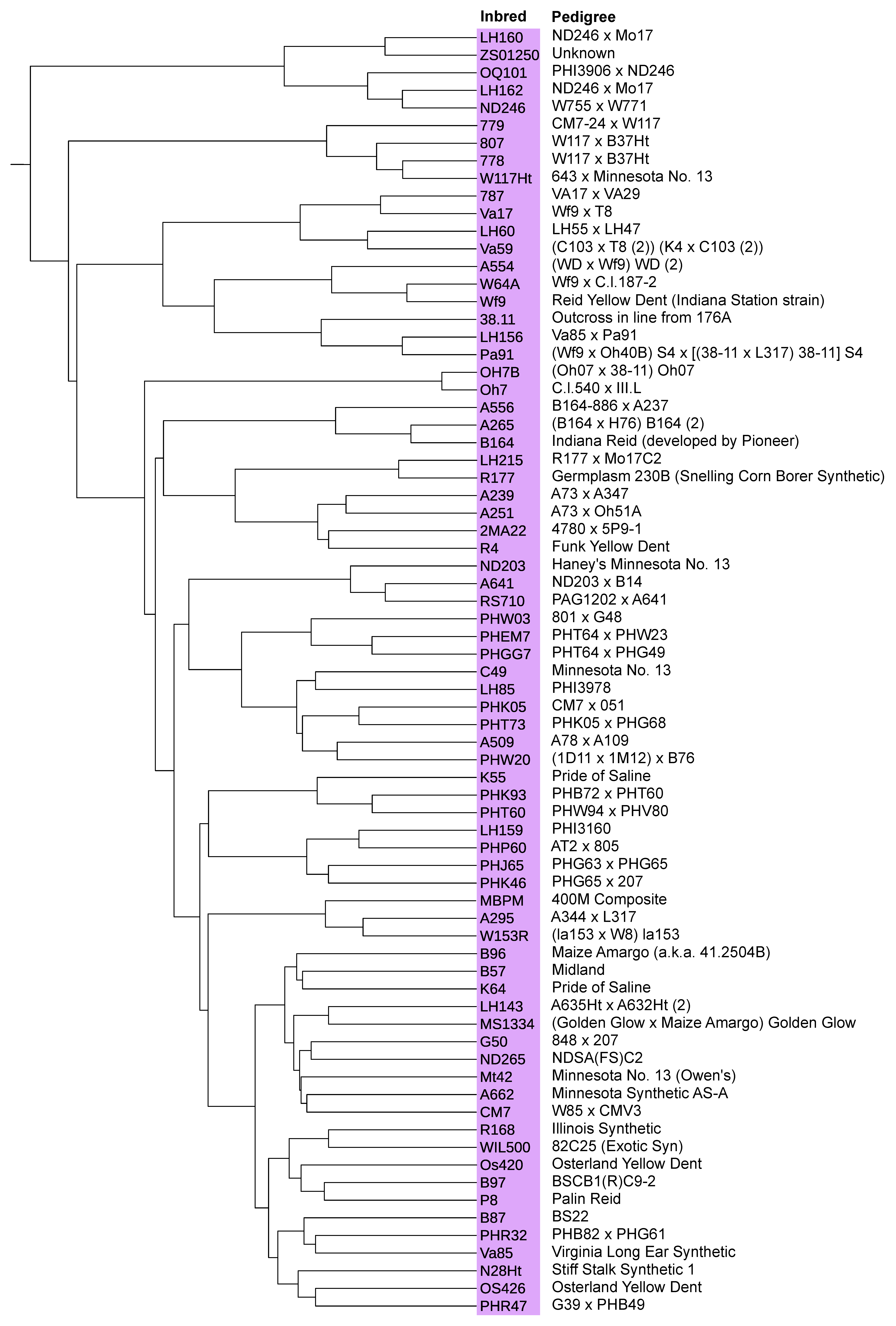

Supplement: S10 Fig — The color surrounding the ex-PVP and public inbred names corresponds with the color assigned to each family subgroup in S1 and S2 Figs. Pedigrees are included to the right of each inbred. PVP inbred pedigrees were obtained from from PVP certificates, available at ars.grin.gov [48]. Public inbred pedigrees were obtained from Gerdes et al., (1993) [49] and Cross et al., (1989) [67]. Consultation of pedigrees, as well as previous publications on the subject [12, 20, 24], confirm individual heterotic group memberships are accurate. (TIFF) [file pone.0189277.s014.tiff]
